# Pigs vs people: the use of pigs as analogues for humans in forensic entomology and taphonomy research

**DOI:** 10.1007/s00414-019-02074-5

**Published:** 2019-06-17

**Authors:** Szymon Matuszewski, Martin J. R. Hall, Gaétan Moreau, Kenneth G. Schoenly, Aaron M. Tarone, Martin H. Villet

**Affiliations:** 1 Laboratory of Criminalistics, Adam Mickiewicz University, Św. Marcin 90, 61-809 Poznań, Poland; 2 Department of Life Sciences, Natural History Museum, Cromwell Road, London, SW7 5BD UK; 3 Département de biologie, Pavillon Rémi-Rossignol, Université de Moncton, Moncton, New Brunswick E1A 3E9 Canada; 4 Department of Biological Sciences, California State University, Stanislaus, 1 University Circle, Turlock, CA 95382 USA; 5 Department of Entomology, Texas A&M University, 2475 TAMU, College Station, TX 77843 USA; 6 Southern African Forensic Entomology Research Laboratory, Rhodes University, Makandha, 6140 South Africa

**Keywords:** Forensic entomology, Forensic taphonomy, Pig carcasses, Human corpses, Animal models, Decomposition ecology

## Abstract

Most studies of decomposition in forensic entomology and taphonomy have used non-human cadavers. Following the recommendation of using domestic pig cadavers as analogues for humans in forensic entomology in the 1980s, pigs became the most frequently used model cadavers in forensic sciences. They have shaped our understanding of how large vertebrate cadavers decompose in, for example, various environments, seasons and after various ante- or postmortem cadaver modifications. They have also been used to demonstrate the feasibility of several new or well-established forensic techniques. The advent of outdoor human taphonomy facilities enabled experimental comparisons of decomposition between pig and human cadavers. Recent comparisons challenged the pig-as-analogue claim in entomology and taphonomy research. In this review, we discuss in a broad methodological context the advantages and disadvantages of pig and human cadavers for forensic research and rebut the critique of pigs as analogues for humans. We conclude that experiments using human cadaver analogues (i.e. pig carcasses) are easier to replicate and more practical for controlling confounding factors than studies based solely on humans and, therefore, are likely to remain our primary epistemic source of forensic knowledge for the immediate future. We supplement these considerations with new guidelines for model cadaver choice in forensic science research.

**Electronic supplementary material:**

The online version of this article (10.1007/s00414-019-02074-5) contains supplementary material, which is available to authorized users.

“We are unlikely to ever know everything about every organism. Therefore, we should agree on some convenient organism(s) to study in great depth, so that we can use the experience of the past (in that organism) to build on in the future. This will lead to a body of knowledge in that ‘model system’ that allows us to design appropriate studies of nonmodel systems to answer important questions about their biology” [1].

“Model species are usually easy to rear, observe, or otherwise experimentally manipulate. They therefore allow knowledge to be built up rapidly and efficiently, because confounding factors are known and thus can be controlled in subsequent experiments” [2].

## Introduction

While collaborating with medical examiners in the late 1800s, French entomologist Pierre Mégnin [3] advanced the first formal definition and testable mechanism of ecological succession and recognized the predictability of carrion-arthropod succession and resource partitioning in human corpses and their application in forensic analysis [4, 5]. These investigations gave birth to the twin disciplines of carrion ecology and forensic entomology. Subsequently, most studies of vertebrate decomposition used non-human carcasses ranging in size from amphibians to elephants (Table 1). Payne innovatively used pig cadavers in his ground-breaking ecological experiments on decomposition [6–8]. Wider interest in forensic entomology and taphonomy arose in the mid-1980s, and such studies initially focussed on pigs or rabbits (Table 1). By the late 1980s, the domestic pig was being recommended as an analogue for humans in forensic entomology research and training workshops [9–11]. Starting in the early 1990s, field studies and statistical models were proposed to test different aspects of the pig-as-analogue claim in forensic entomology [12–14].

**Table 1:** Selected cadaver studies in carrion ecology, forensic entomology and taphonomy. References to this table are listed in Electronic Supplementary Material

Author(s)	Date of publication	Locality	Animal model	Major research focus
Chapman and Sankey [1]	1955	England	Rabbits	Arthropod inventory; habitats
Bornemissza [2]	1957	Australia	Guinea pig	Arthropod inventory; succession
Reed [3]	1958	USA	Dogs	Arthropod inventory; succession
Payne [4]	1965	USA	Pigs	Surface decomposition; insect access
Payne et al. [5]	1968	USA	Pigs	Underground decomposition
Payne and King [6]	1972	USA	Pigs	Water decomposition
Nabagło [7]	1973	Poland	Bank voles	Surface/underground decomposition; insect inventory; succession; seasons
Cornaby [8]	1974	Costa Rica	Lizards, toads	Arthropod inventory; succession; habitats
Johnson [9]	1975	USA	Small mammals	Arthropod inventory; succession; seasons
Smith [10]	1975	England	Fox	Arthropod inventory; succession
Coe [11]	1978	Kenya	Elephants	Surface decomposition; insect inventory
McKinnerney [12]	1978	USA	Rabbits	Arthropod inventory; succession; scavenging
Jiron and Cartin [13]	1981	Costa Rica	Dogs	Arthropod inventory; succession
Abell et al. [14]	1982	USA	Turtles	Arthropod inventory; succession
Rodriguez and Bass [15]	1983	USA	Humans	Insect inventory; succession
Schoenly and Reid [16]	1983	USA	Various mammals	Cadaver mass; insect inventory
Lord and Burger [17]	1984	USA	Gulls	Arthropod inventory; succession; seasons; habitats; scavenging
Rodriguez and Bass [18]	1985	USA	Humans	Underground decomposition
Early and Goff [19]	1986	Hawaii	Cats	Surface decomposition; arthropod inventory; succession
Micozzi [20]	1986	USA	Rats	Freezing; wounds
Braack [21]	1986	South Africa	Impala	Insect inventory
Peschke et al. [22]	1987	Germany	Rabbits	Insect inventory; succession; habitats; seasons
Tullis and Goff [23]	1987	Hawaii	Pig	Surface decomposition; arthropod inventory; succession
Blacklith and Blacklith [24]	1990	Ireland	Birds, mice	Insect inventory; habitats
Kentner and Streit [25]	1990	Germany	Rats	Insect inventory; succession; habitats
Hewadikaram and Goff [26]	1991	Hawaii	Pigs	Cadaver mass
Vass et al. [27]	1992	USA	Humans	Compounds released into soil during decomposition
Shean et al. [28]	1993	USA	Pigs	Sun exposure
Anderson and VanLaerhoven [29]	1996	Canada	Pigs	Insect inventory; succession
Tantawi et al. [30]	1996	Egypt	Rabbits	Insect inventory; succession; seasons
Keiper et al. [31]	1997	USA	Rats	Water decomposition; habitats; arthropod inventory
Richards and Goff [32]	1997	Hawaii	Pigs	Arthropod inventory; succession; habitats
Avila and Goff [33]	1998	Hawaii	Pigs	Burnt cadaver decomposition; habitats; succession
Komar and Beattie [34, 35]	1998	Canada	Pigs	Cadaver mass; habitats; clothing; post-mortem artefacts
Tomberlin and Adler [36]	1998	USA	Rats	Water decomposition; insect inventory; seasons; habitats
Bourel et al. [37]	1999	France	Rabbits	Insect inventory; succession; habitats
DeJong and Chadwick [38]	1999	USA	Rabbits	Insect inventory; succession; habitats
Turner and Wiltshire [39]	1999	England	Pigs	Underground decomposition
VanLaerhoven and Anderson [40]	1999	Canada	Pigs	Underground decomposition; insect inventory; succession; habitats
Carvalho et al. [41]	2000	Brazil	Pigs, humans	Insect inventory
Davis and Goff [42]	2000	Hawaii	Pigs	Intertidal habitats; succession
Shalaby et al. [43]	2000	Hawaii	Pigs	Hanging cadaver decomposition; succession
Arnaldos et al. [44]	2001	Spain	Chickens	Insect inventory; succession
Carvalho and Linhares [45]	2001	Brazil	Pigs	Insect inventory; succession
Marchenko [46]	2001	Russia	Dogs, cats, rabbits, pigs	Decomposition in various scenarios; seasons; habitats; insect repellents; clothing, plant response to cadavers
Wolff et al. [47]	2001	Colombia	Pigs	Insect inventory; succession
Yan et al. [48]	2001	USA	Pigs	Adipocere formation
Centeno et al. [49]	2002	Argentina	Pigs	Insect inventory; seasons; habitats; succession
Hobischak and Anderson [50]	2002	Canada	Pigs	Water decomposition; habitat; arthropod inventory; succession
LeBlanc and Strongman [51]	2002	Canada	Pigs	Insect inventory; habitats
Archer and Elgar [52, 53]	2003	Australia	Pigs	Insect inventory; seasons; colonisation patterns
Bharti and Singh [54]	2003	India	Rabbits	Insect inventory; seasons; succession
Kočárek [55]	2003	Czech Republic	Rats	Insect inventory; seasons; habitats; succession
Shahid et al. [56]	2003	USA	Pigs	Arthropod saturation in human taphonomy facilities
Watson and Carlton [57–59]	2003, 2005	USA	Bear, deer, alligators, pigs	Insect inventory; seasons; succession; animal models comparison
Anderson and Hobischak [60]	2004	Canada	Pigs	Marine decomposition
Archer [61, 62]	2004	Australia	Pigs	Succession; seasons; annual variation; abiotic determinants of decomposition rate
Arnaldos et al. [63]	2004	Spain	Chickens	Insect inventory; seasons; succession
Grassberger and Frank [64]	2004	Austria	Pigs	Urban decomposition; insect inventory; succession
Tabor et al. [65, 66]	2004, 2005	USA	Pigs	Insect inventory; succession; seasons
Vass et al. [67]	2004	USA	Humans	Volatiles of decomposition
Anderson [68]	2005	Canada	Pigs	Arson and insect evidence
Moura et al. [69]	2005	Brazil	Rats	Succession mechanisms; seasons; habitats
Perez et al. [70]	2005	Colombia	Pigs	Urban decomposition; insect inventory; succession
Schoenly et al. [71]	2005	USA	Pigs	Arthropod saturation in human taphonomy facilities
Weitzel [72]	2005	Canada	Pigs	Underground decomposition; seasons
DeJong and Hoback [73]; DeJong et al. [74]	2006; 2011	USA	Rats	Investigator disturbance; insect inventory; succession
Hobischak et al. [75]	2006	Canada	Pigs	Sun exposure; insect inventory; succession
Joy et al. [76]	2006	USA	Pigs	Blow fly inventory; habitats; annual variation; maggot mass
Lang et al. [77]	2006	Australia	Possums	Insect inventory; colonisation patterns
Adlam and Simmons [78]	2007	UK	Rabbits	Repeated cadaver disturbance
Gruner et al. [79]	2007	USA	Pigs	Blow fly inventory; seasons; annual variation
Martinez et al. [80]	2007	Colombia	Pigs	Insect inventory; succession
O’Brien et al. [81]	2007	Australia	Pigs	Scavenging
Schoenly et al. [82]	2007	USA	Pigs, humans	Sampling techniques; human/pig comparison
Benninger et al. [83]	2008	Canada	Pigs	Compounds released into soil during decomposition
Eberhardt and Elliot [84]	2008	New Zealand	Pigs	Insect inventory; succession; habitats
Fiedler et al. [85]	2008	Germany	Pigs	Adult fly inventory; succession; habitats
Huntington et al. [86]	2008	USA	Pigs	Blow fly multigenerational colonisation
Matuszewski et al. [87]	2008	Poland	Pigs	Insect inventory; succession; habitats
Moretti et al. [88]	2008	Brazil	Mice, rats	Insect inventory; succession; seasons
Sharanowski et al. [89]	2008	Canada	Pigs	Insect inventory; succession; seasons; sun exposure
Ururahy-Rodrigues et al. [90]	2008	Brazil	Pigs	Post-mortem artefacts
Voss et al. [91]	2008	Australia	Pigs	Inside-car decomposition; colonisation patterns
Wang et al. [92]	2008	China	Pigs	Insect inventory; succession; seasons
Charabidze et al. [93]	2009	France	Rats, Mice	Insect repellents; colonisation patterns
Dekeirsschieter et al. [94]	2009	Belgium	Pigs	Volatiles of decomposition
Kalinová et al. [95]	2009	Czech Republic	Mice	Carrion beetle attractants
Kelly et al. [96, 97]	2009, 2011	South Africa	Pigs	Wounds; wrapping; clothing
Kjorlien et al. [98]	2009	Canada	Pigs	Scavenging; habitats; clothing
Nelder et al. [99]	2009	USA	Alligators	Succession
Özdemir and Sert [100]	2009	Turkey	Pigs	Insect inventory; succession; seasons
Pakosh and Rogers [101]	2009	Canada	Pigs (limbs)	Water decomposition;
Parmenter and MacMahon [102]	2009	USA	Various mammals and birds	Seasons; surface/underground decomposition; scavenging; nutrient cycling
Segura et al. [103]	2009	Colombia	Pigs	Insect inventory; succession
Van Belle et al. [104]	2009	Canada	Pigs	Compounds released into soil during decomposition; surface/underground decomposition
Voss et al. [105]	2009	Australia	Pigs	Insect inventory; succession; seasons; habitats
Bachmann and Simmons [106]	2010	UK	Rabbits	Underground decomposition; colonisation patterns
Battán Horenstein et al. [107–109]	2010, 2011, 2012	Argentina	Pigs	Insect inventory; succession; seasons; habitats
Bonacci et al. [110]	2010	Italy	Pigs	Insect inventory; seasons; succession
Carter et al. [111]	2010	Australia	Rats	Underground decomposition
Chin et al. [112]	2010	Malaysia	Pigs	Hanging cadaver decomposition
Cross and Simmons [113]	2010	UK	Pigs	Wounds
Matuszewski et al. [114–116]	2010, 2011	Poland	Pigs	Surface decomposition; insect inventory; seasons; habitats; succession
Michaud et al. [117]	2010	Canada	Pigs	Insect inventory; seasons; habitats
Reibe and Madea [118]	2010	Germany	Pigs	Colonisation patterns; habitats
Sabanoglu and Sert [119]	2010	Turkey	Pigs	Insect inventory; succession; seasons
Simmons et al. [120]	2010	UK	Rabbits	Insect access; surface/underground decomposition
Simmons et al. [121]	2010	UK	Pigs	Insect access; cadaver mass
Swann et al. [122, 123]	2010	Canada, Australia	Pigs	Compounds released during decomposition
Szpila et al. [124]	2010	USA, Poland	Pigs, rats	Colonisation of buried cadavers
Valdes-Perezgasga et al. [125]	2010	Mexico	Pigs	Insect inventory; succession
Ahmad et al. [126]	2011	Malaysia	Macaques	Wrapping
Anderson [127]	2011	Canada	Pigs	Indoor/outdoor decomposition
Anton et al. [128]	2011	Germany	Pigs	Insect inventory; succession; seasons
Barrios and Wolff [129]	2011	Colombia	Pigs	Water decomposition; arthropod inventory; succession; habitats
Bajerlein et al. [130]	2011	Poland	Pigs	Seasons; habitats; colonisation patterns
Bugajski et al. [131]	2011	USA	Pigs	Freezing
Cassar et al. [132]	2011	Australia	Pigs	Adipocere formation
DeVault et al. [133]	2011	USA	Mice	Scavenging
Dickson et al. [134]	2011	New Zealand	Pigs (heads)	Marine decomposition; bacterial succession
von Hoermann et al. [135]	2011	Germany	Pigs	Hide beetle attractants
Spicka et al. [136]	2011	USA	Pigs	Cadaver mass
Statheropoulos et al. [137]	2011	Greece	Pigs	Volatiles of decomposition
Voss et al. [138]	2011	Australia	Pigs	Clothing
Al-Mesbah et al. [139]	2012	Kuwait	Rabbits	Insect inventory; habitats; succession
Brasseur et al. [140]	2012	Belgium	Pigs	Volatiles of decomposition
Gruenthal et al. [141]	2012	UK	Pigs	Burnt cadaver decomposition
Martin-Vega and Baz [142, 143]	2012, 2013	Spain	Squids	Carrion and skin beetle inventory; seasons; habitats
Ortloff et al. [144]	2012	Chile	Pigs	Insect inventory; succession
Prado e Castro et al. [145, 146]	2012, 2013	Portugal	Pigs	Insect inventory; succession; seasons
Shelomi et al. [147]	2012	USA	Pigs	Insect repellents; blow fly colonisation patterns
Stadler et al. [148]	2012	Canada	Pigs	Volatiles of decomposition
Widya et al. [149]	2012	UK	Rabbits	Water decomposition; adipocere formation
Azwandi et al. [150]	2013	Malaysia	Rats, rabbits, macaques	Insect inventory; succession; rat/rabbit/monkey comparison
Barton et al. [151]	2013	Australia	Kangaroos	Carrion and biodiversity
Benbow et al. [152]	2013	USA	Pigs	Insect inventory; succession; seasons
Bygarski and LeBlanc [153]	2013	Canada	Pigs	Insect inventory; succession
Dekeirsschieter et al. [154]	2013	Belgium	Pigs	Rove beetle inventory; seasons
von Hoermann et al. [155]	2013	Germany	Pigs	Carrion beetle attractants
Hyde et al. [156]	2013	USA	Humans	Cadaver microbiome
Johansen et al. [157]	2013	Norway	Mice	Blow fly attractants
Johnson et al. [158]	2013	Australia	Pigs	Thermogenesis in cadavers
Lowe et al. [159]	2013	Canada	Pigs	Textiles degradation on buried cadavers
Matuszewski et al. [160]; Mądra et al. [161]	2013, 2014	Poland	Pigs	Insect inventory; habitats; seasons
Metcalf et al. [162]	2013	USA	Mice	Cadaver microbiome
Meyer et al. [163]	2013	USA	Pigs	Surface decomposition; seasons
Sutherland et al. [164]	2013	South Africa	Pigs	Cadaver mass
von der Luhe [165]	2013	Canada	Pigs	Compounds released into soil during decomposition
Abouzied [166]	2014	Saudi Arabia	Rabbits	Insect inventory; seasons; succession
Anderson and Bell [167]	2014	Canada	Pigs	Marine decomposition; arthropod inventory
Bhadra et al. [168]	2014	England	Pigs (heads)	Colonisation patterns
Caballero and León-Cortéz [169]	2014	Mexico	Pigs	Beetle inventory; succession; habitats
Corrêa et al. [170]	2014	Brazil	Rabbits	Beetle inventory; seasons
Farwig et al. [171]	2014	Germany	Mice	Biotic determinants of decomposition rate; seasons
Matuszewski et al. [172, 173]; Mądra et al. [174]	2014, 2016, 2015	Poland	Pigs	Cadaver mass; clothing; insect inventory; long-term decomposition
Mohr and Tomberlin [175, 176]	2014, 2015	USA	Pigs	Cadaver visitation by adult blow flies
Oliveira-Costa et al. [177]	2014	Brazil	Pigs	Succession on burnt cadavers
Pechal et al. [178]	2014	USA	Pigs	Delayed insect access; colonisation patterns; succession
Pechal et al. [179]	2014	USA	Pigs	Cadaver microbiome
Perrault et al. [180–182]	2014, 2015	Australia	Pigs	Volatiles of decomposition
Whitaker [183]	2014	USA	Pigs, humans	Pig/human comparison of blow fly colonisation
Young et al. [184]	2014	England	Deer	Scavenging
Zurawski et al. [185]	2014	USA	Pigs	Nocturnal blow fly oviposition
Agapiou et al. [186]	2015	Greece	Pigs	Volatiles of decomposition
Alexander et al. [187]	2015	USA	Humans	Residual odour of decomposition in the soil
Aubernon et al. [188]	2015	France	Rats	Blow fly development on contaminated cadaver
Baz et al. [189]	2015	Spain	Squids	Insect inventory; habitats
Card et al. [190]	2015	England	Pigs	Clothing
Farrell et al. [191]	2015	Australia	Pigs	Insect inventory
Hyde et al. [192]	2015	USA	Humans	Cadaver microbiome
Iancu et al. [193]	2015	Romania	Pigs	Insect and microbe inventory; succession
Iancu et al. [194]	2015	Romania	Pigs	Insect and microbe inventory; succession
Lynch-Aird et al. [195]	2015	England	Pigs	Hanging cadaver decomposition
Martin-Vega et al. [196]	2015	Spain	Squids	Clown beetle inventory; habitats
Paczkowski et al. [197]	2015	Germany	Pigs	Volatiles of decomposition
Roberts and Dabbs [198]	2015	USA	Pigs	Freezing
Rysavy and Goff [199]	2015	Hawaii	Pigs	Underground decomposition; insect inventory
Silahuddin et al. [200]	2015	Malaysia	Rabbits	Insect inventory; succession; habitats
Stadler et al. [201]	2015	Canada	Pigs	Volatiles of decomposition
Sukchit et al. [202]	2015	Thailand	Pigs	Insect inventory; habitats; succession; seasons; hanging; clothing
Szpila et al. [203]	2015	Poland	Pigs	Insect inventory; succession
Ueland et al. [204]	2015	Australia	Pigs	Textiles degradation on surface cadavers
Zanetti et al. [205, 206]	2015	Argentina	Pigs	Underground decomposition; beetle inventory; seasons
Zeariya et al. [207]	2015	Egypt	Rabbits, dogs	Insect inventory; succession; habitats
Anderson and Bell [208]	2016	Canada	Pigs	Marine decomposition; seasons
Cammack et al. [209]	2016	USA	Pigs	Concealment; seasons
Lyu et al. [210]	2016	China	Pigs	Beetle inventory
Mashaly [211]	2016	Egypt	Rabbits	Burnt cadaver decomposition; insect inventory; succession; habitats
Metcalf et al. [212]	2016	USA	Mice, Humans	Cadaver microbiome
Moffatt et al. [213]	2016	England	Pigs	Distribution of maggots length on carrion
Parry et al. [214]	2016	South Africa	Fishes	Fly inventory; habitats; seasons
Perez et al. [215]	2016	USA	Pigs	Distance between cadavers
Weidner et al. [216]	2016	USA	Pigs	Blow fly colonisation timing
Weiss et al. [217]	2016	USA	Pigs	Cadaver microbiome
Vasconcelos et al. [218]	2016	Brazil	Pigs	Fly inventory
Amendt et al. [219]	2017	Germany	Pigs	Thermal imaging of cadavers
Connor et al. [220]	2017	USA	Pigs, humans	Human/pig comparison
Fancher et al. [221]	2017	USA	Humans	Compounds released into soil during decomposition
Marais-Werner et al. [222]	2017	South Africa	Pigs	Underground decomposition
Martin-Vega et al. [223]	2017	Spain	Pigs	Colonisation patterns; seasons
Mashaly [224]	2017	Saudi Arabia	Rabbits	Beetle inventory; habitats; succession
McIntosh et al. [225]	2017	Australia	Pigs	Burnt cadaver decomposition; succession
Michaud and Moreau [226]	2017	Canada	Pigs	Succession mechanisms
Niederegger et al. [227]	2017	Germany	Pigs	Wounds
Pacheco et al. [228]	2017	Canada	Pigs	Blow fly colonisation patterns
Roberts et al. [229]	2017	USA	Humans	Cadaver mass
Scholl and Moffatt [230]	2017	England	Pigs	Dismemberment; concealment in plastic sacks
Wang et al. [231]	2017	China	Pigs, humans, rabbits	Human/pig/rabbit comparison; surface decomposition; succession;
Wang et al. [232]	2017	China	Pigs	Exposure daytime; succession;
Weidner et al. [233]	2017	USA	Pigs	Comparison of bait traps and cadaver inventories
Cruise et al. [234, 235]	2018	USA	Pigs	Insect inventory; succession; sampling techniques
Dautartas et al. [236]; Steadman et al. [237]	2018	USA	Pigs, humans, rabbits	Human/pig/rabbit comparison; surface decomposition; scavenging
Díaz-Aranda et al. [238]	2018	Spain	Pigs	Insect inventory; succession; seasons
Frątczak-Łagiewska and Matuszewski [239]	2018	Poland	Pigs	Silphid beetles; succession; seasons; habitats
von Hoermann et al. [240]	2018	Germany	Pigs	Carrion beetle inventory; habitats
Knobel et al. [241]	2018	Australia	Pigs, humans	Decomposition rates; odour profiles; human/pig comparison
Lee et al. [242]	2018	Australia	Pigs	Thermal imaging of cadavers
Lutz et al. [243]	2018	Canada	Pigs	Beetle colonisation and breeding on concealed carcasses
Mañas-Jordá et al. [244]	2018	Mexico	Pigs	Fly inventory; succession; habitats
Marais-Werner et al. [245]	2018	South Africa	Pigs	Surface/underground decomposition
Pérez-Marcos [246]	2018	Spain	Pigs, chickens	Fly inventory; pig/chicken comparison
Salimi et al. [247]	2018	Iran	Rabbits	Insect inventory; succession; seasons; habitats
Shayya [248]	2018	Lebanon	Pigs	Clown beetle inventory; succession; seasons; habitats
Singh et al. [249]	2018	USA	Humans	Arthropod and microbe inventory and succession in the soil below a cadaver
Spies et al. [250, 251]	2018	South Africa	Pigs	Scavenging
Szelecz et al. [252, 253]	2018	Switzerland	Pigs	Compounds released into soil during decomposition; clown beetle colonisation of hanging and surface cadavers

**Fig. 1:**
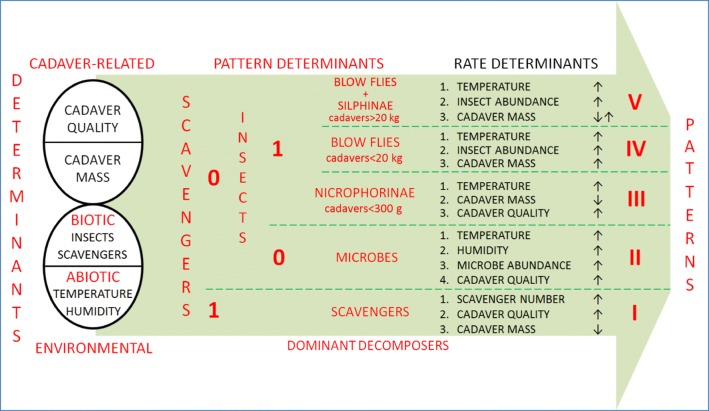
Determinants and general patterns of cadaver decomposition—synthesis based on findings of cadaver decomposition studies (Table 1). Numbers I–V denote general patterns of decomposition (differing according to dominant decomposers, key determinants of decomposition rate and the effect they have on decomposition). Numbers “0” and “1” denote absence and presence of scavengers or insects. Arrows next to rate determinants indicate whether a determinant, considered in isolation, is positively (↑) or negatively (↓) related to decomposition rate. Some determinants in this figure should be considered as sets of simple determinants, e.g. cadaver quality including body mass index, antemortem cadaver modifications (e.g. pharmaceuticals use), postmortem modifications (e.g. freezing during the winter) and others

Examples of taphonomic studies have been cited from as far back as Leonardo da Vinci in the fifteenth century, but the field began to achieve formality in the 1940s [15]. In the 1970s, palaeoanthropology used taphonomy to interpret the deposition of hominid remains in fossil-rich sites, particularly to provide information about how the hominids lived and died [16, 17]. Integration of fossil-focused taphonomy with physical anthropology led to the differentiation of forensic taphonomy, which relied on extensive comparisons of palaeontological, archaeological and modern case studies [18]. The development of pigs as model organisms in forensic entomology provided a more experimental approach for forensic taphonomy and established some major patterns regarding vertebrate decomposition (Table 1, Fig. 1).

The advent of outdoor human taphonomy facilities (often mistermed “body farms” [19]) facilitated experimental studies using human cadavers. First amongst these was the University of Tennessee Anthropological Research Facility, while the first outside the USA was the Australian Facility for Taphonomic Experimental Research (AFTER) [19, 20]. At least eight facilities now exist, six in the USA, one in Australia and one in the Netherlands [20–22]. The facilities have allowed experimental comparison of decomposition in human and non-human models under a variety of conditions [14, 23, 24]. Since then, debate has arisen over the relevance of taphonomic studies for forensics (e.g. [19, 25, 26]), and the proper associated experimental (and ethical) protocols [27, 28]. There is variation in the source populations contributing to taphonomy facilities; moreover, their source cadavers (usually elders dying of natural causes) systematically differ from cadavers involved in forensic scenarios (usually adults dying of unnatural causes). Therefore, for a variety of reasons, the findings from these facilities may be difficult to extrapolate to other human populations and to typical forensic cases.

Recent publications have raised the opportunity to consolidate what has been learned from animal models in decomposition studies, and to examine the implications of this knowledge for the design of field experiments in forensic entomology and taphonomy, specifically, whether animal carcasses can effectively substitute for human cadavers, which is the major aim of this review. Our major focus is research on principles concerning cadaver decomposition, including the associated arthropods and their succession. Therefore this paper does not extensively address topics related to the accuracy and precision of PMI estimation techniques developed in forensic entomology or taphonomy.

## Lessons from pig cadavers

The use of animal models to advance knowledge dates back to the ancient Greek times with dogs and chicks used to study human anatomy, physiology and ontogeny [29]. Nowadays, animal models are used to study a large array of human related-issues, e.g. diseases [30], mental and neuropsychiatric disorders [31] or orthopaedic and dental implants [32]. In a similar way, our current understanding of animal decomposition is largely derived from experiments with non-human cadavers, with pig carcasses contributing overwhelmingly to this knowledge (Table 1). Payne’s [6] experimental work using piglets was a watershed event in carrion ecology for its impact and originality. After trying carrion from different vertebrate animals (amphibians, mammals, birds), Payne settled on domesticated pigs because he knew the time of their death, he could acquire them in large numbers of uniform age and mass, and their relatively hairless skin and lack of feathers made insect sampling easier than from alternative carcasses. In his experiments, Payne used cages with different mesh sizes to provide open and limited access to insects to document daily changes in carcass decay and dismemberment. He found that carcasses protected from insects mummified, remaining intact for months; whereas, carcasses exposed to insects lost 90% of their starting mass in just 6 days. This result showed that insect access is a key determinant of cadaver decay.

Inspired by Payne’s experimental protocol, forensic entomologists started using pig cadavers in studies focused on inventorying carrion-arthropod faunas and successional patterns, which have been described for a long list of countries and habitats (Table 1). Although the species involved varied between biogeographical regions, ecological guilds were consistent and functioned in a very consistent way (Table 1). Pigs have illustrated patterns of decomposition over timescales of days, seasons and years (Table 1). Seasonal components of variation in the insect community are relatively well understood and several quantitative models have been proposed to describe the ecological succession that occurs in the arthropod community on a cadaver (Table 1). Much of the early work followed the stage-based paradigm (e.g. [6]). Decay stages, named according to physiochemical changes seen in the cadaver, accompanied timetables of insect succession. Stage descriptions varied in both number and duration; moreover, the widely-held view was that the onset of each stage was marked by an abrupt change in the insect community, similar to Mégnin’s [3] notion of “squads”. Subsequent ecological and forensic studies found that succession in carrion largely follows a continuum of gradual changes [33–35]. Despite these findings, the use of stages of decomposition is still frequent in the forensic literature [35].

More recently, pigs became model animals in experimental research of forensic entomology and taphonomy (Table 1). Pigs have influenced recent theoretical developments in carrion and succession ecology and shaped our understanding of how vertebrate cadavers decompose in various environments, including indoor, suspended, buried, epigeic, intertidal, marine and freshwater settings. A wide spectrum of habitats has been investigated (Table 1) and found to show some idiosyncratic variations on otherwise very general patterns (Fig. 1). Results of these studies indicate that temperature and access or abundance of carrion insects are key environmental determinants of cadaver decomposition, whereas cadaver mass is a key cadaver-related determinant (Table 1, Fig. 1). At least five general decomposition patterns may be currently discerned: decay driven by either vertebrate scavengers, microbes, burying beetles, blow flies or blow flies with silphid beetles, with distinct key determinants of decomposition rate in each of the patterns (Table 1, Fig. 1).

Human cadavers vary in many characteristics that influence decomposition, most of which have been investigated using pigs (Table 1). Pre- or postmortem modifications such as wounds, burning, wrapping, dismemberment, contamination, concealment and clothing may affect the colonisation process and eventually decomposition to varying degrees, depending on their intensity and context of action (Table 1). Some modifications do not affect the whole cadaver, leaving parts of it to be colonized by insects in their usual manner, while other modifications such as clothing have effects on insect colonisation or succession that are too small or too variable to have practical consequences for estimates of postmortem intervals (PMIs). Other modifications delay colonisation by insects but have little consequence once colonisation has occurred. The same modifications may however differently affect non-entomological processes, for example, clothing influences rate of cadaver cooling and therefore is considered important for some pathology-based methods for estimation of PMI, e.g. Henssge’s nomogram method [36]. Regarding insects, the implications of modification appear to be larger than the effect of the cadaver’s species.

In parallel, pig cadavers were used to test new forensic techniques or validate well-established ones (Table 2). They have provided proof-of-concept for techniques as simple as entomological sampling and as sophisticated as ground penetrating radar or thermal imaging to locate cadavers (Table 2). Many of these techniques have gone on to be applied to forensic investigations involving humans, demonstrating in this way the practicality of pigs as model cadavers.

**Table 2:** Forensic methods and techniques developed, refined or tested using pig cadavers. References to this table are listed in Electronic Supplementary Material

Method/technique	References	Pig cadaver use
Field protocol for experimental studies on PMI	Schoenly et al. [1, 2]	Tests of the protocol
Model organisms	Watson and Carlton [3, 4]	Comparisons of different animals
	Schoenly et al. [2]; Wang et al. [5]; Connor et al. [6]; Dautartas et al. [7]	Comparisons of pigs and humans
Human-size insect trap for studying succession	Schoenly et al. [1]	Recorded trap microclimate and carrion-arthropod families caught by trap
Device for sampling cadaver-related aquatic insects	Vance et al. [8]	Tests of trap efficiency in catching aquatic insects
Degree-day index for decomposition related processes	Michaud and Moreau [9]	Development of the index and tests for its reliability
Reconstruction of temperature conditions	Hofer et al. [10]	Reliability of temperature recordings on a death scene
Temperature methods for insect pre-appearance interval (PAI)	Matuszewski [11, 12]	Development of PAI models; tests of the method
	Matuszewski and Szafałowicz [13]; Archer [14]; Matuszewski et al. [15]	Development of PAI models
	Matuszewski and Mądra 2015 [16]	Tests of the protocols for PAI field studies
	Matuszewski and Mądra-Bielewicz [17]	Validation of PAI methods
Total body score	Myburgh et al. [18]	Validation of the method
	Lynch-Aird et al. [19]	Development of TBS for hanging cadavers
	Nawrocka et al. [20]	Inter-rater reliability of the TBS
	Keough et al. [21]	Amendment of TBS for pig cadavers
	Ribéreau-Gayon et al. [22]	Reliability of TBS based on cadaver pictures
PMI estimation based on insect succession	Michaud and Moreau [23]	Tests of predictability of insect occurrence based on degree-day accumulation
	Michaud and Moreau [24]	Tests of sampling protocols for field studies
	Perez et al. [25]	Evaluation of utility of insect taxa for derivation of confidence intervals about PMI estimate
	Mohr and Tomberlin [26]	Tests of oocyte development of adult blow flies visiting cadaver as a PMI indicator
	Perez et al. [27]	Tests of minimum inter-cadaver distances for forensic field studies
	Matuszewski [28]	Tests of presence/absence of insect taxa as an approach for PMI estimation
	Mądra-Bielewicz et al. [29]	Tests of insect sex and size as PMI indicators
	Cruise et al. [30]	Tests of the protocols for cadaver field studies
PMI estimation based on insect development	VanLaerhoven [31]	Validation of methods
	Reibe-Pal and Madea [32]	Comparison of methods
	Weatherbee et al. [33]	Validation of methods
PMI estimation based on microbes	Pechal et al. [34]	Tests of usefulness of microbe succession for PMI estimation
Exposed cadavers searching	Amendt et al. [35]; Lee et al. [36]	Tests of thermal imaging techniques used from the air
Clandestine burial searching	Schultz et al. [37]; Schultz [38]; Salsarola et al. [39]	Tests of ground-penetrating radar
Submerged cadavers searching	Healy et al. [40]	Tests of side-scan sonar
Detection of gasoline in cadaver tissues	Pahor et al. [41]	Proof-of-concept tests

## Are pigs an appropriate model for forensic entomology and taphonomy?

A comparison of the advantages and disadvantages of pig and human cadavers for experimental forensic entomology and taphonomy research (Table 3) indicates that pigs are usually superior to humans in such experiments. Most importantly, pig cadavers may easily be replicated in large numbers and at low cost, whereas access to human corpses is restricted to taphonomic facilities or medical examiner’s offices with all of their associated inherent difficulties. At taphonomic facilities, waiting times for receiving replicate bodies on multiple-donation days are unpredictable and uncontrollable [37], even if minimum criteria are met for accepting cadavers as “replicates” (i.e. death within 48 h of acquisition, intact, unautopsied, unembalmed and refrigerated). The difficulty in amassing replicate human cadavers allows little experimental control over key decomposition determinants such as cadaver mass. The unpredictable and uncontrollable variation inherent in cadaver availability may limit the value of observations in humans and invalidate the experiment, by producing statistically underpowered comparisons that are insufficient to detect significant differences and by enlarging the risk of confounding effects. In addition, the practical realities of working with human remains can limit the types of information that can be gleaned from and about them. Moreover, the continual association of the taphonomy facilities with human cadavers can itself present a challenge. Although a 1998 field study at the Tennessee facility found little evidence of cadaver enrichment effects on the surface-active entomofauna or decay rates using pig carcasses [38, 39], a recent study of soil parameters [40] demonstrated that the Tennessee site is contaminated with high levels of decomposition products, which may limit the interpretation of certain nutrient-based taphonomic results as no reliable baseline sample can be obtained within the facility.

**Table 3:** Advantages and disadvantages of domestic pig and human cadavers in forensic entomology and taphonomy research related to human decomposition [6, 9–11, 14, 23, 24, 44, 65]

	Pig cadavers	Human cadavers
Cons	1. Dissimilar to human cadavers in some important aspects: a. Body proportions b. Gastrointestinal anatomy c. Diet (more uniform, larger proportion of plant products) 2. More uniform than humans 3. Unacceptable in some cultures	1. Difficult to replicate: a. Available in low numbers b. Time and cause of death beyond researcher control (self-donation, age, disease incidence etc.) c. Dissimilar to each other in: • Mass • Age, sex, ethnicity • Antemortem pharmaceuticals use • Body conditions (frozen/fresh, autopsied/non-autopsied, etc.) 2. Limitations of taphonomy facilities (body farms): a. Small area, potential for insufficient inter-cadaver distances b. Uniform abiotic conditions c. Frequently non-natural conditions d. Area saturated with cadavers 3. Limitations of casework (i.e. medical examiner samples): a. Restricted to observation b. Cannot control effects of routine processing of remains c. Sometimes no information about death circumstances and the cadaver itself 4. Risk of sensationalized research a. Complex ethical considerations/generally unacceptable b. Potentially negative publicity c. Potential for findings to be “oversold”
Pros	1. Similar to human cadavers in some important aspects: a. Body mass range b. Anatomy c. Body composition d. Skin coverage with hair e. Gut microbiota f. Gross processes of decay 2. Easy to replicate: a. Cheap and available in large numbers b. Time and cause of death controllable c. Cadaver traits controllable d. Possible to work with unfrozen cadavers 3. Less sensationalized research and relatively straightforward ethical considerations	1. No species-related differences

While, in many cases, researchers may be interested in how the decomposition process works in humans, the available human remains are either derived from inappropriate populations, cannot be linked to control samples or are too variable for robust experiments. Due to these practicalities, pig cadavers are usually the best choice available for most experimental purposes in forensic sciences. Moreover, pig cadavers may be used to compare treatments of relevance with forensic scenarios and to make inferences about human decomposition. If treatment A results in a slower decomposition than treatment B in pigs, in the absence of other information, we can reasonably assume a similar effect in humans, especially if it can be supported with other knowledge and logic. The possibility that a model animal and the humans that it models decompose differently does not make that model useless; it depends on the specific question being addressed. This conclusion has much wider applicability. For example, mouse cadavers were useful in demonstrating forensic applications of microbiology [41, 42]. Postmortem microbiome comparisons between different animals revealed the common appearance of some informative bacterial taxa across rodent, pig and human models [41–43]. Another example is the use of rabbit cadavers to provide local carrion insect inventories (Table 1). When early cadaver colonizers (e.g. blow flies) are the focus, rabbits are as informative as pigs or humans, but when middle or late colonizers (e.g. beetles of Silphinae or Cleridae) are studied, rabbit cadavers are inappropriate, because such insects rarely colonize carcasses as small as rabbits [44, 45].

Comparative studies of pig and human cadavers revealed largely overlapping insect faunas [14, 44], with as much difference between individual pigs or humans as between pigs and humans [46]. Similarly, insect faunas compiled from human case studies (e.g. [47, 48]) largely resembled those from pig cadaver experiments (Table 1). Although alligator carrion revealed important faunal differences compared with large mammals (i.e. pigs, bears and deer), the latter group yielded highly similar insect community composition [49, 50]. These results indicate that, when compared across related cadaver taxa of similar size, carrion insects (i.e. necrophagous insects) show negligible preference for one cadaver taxon over another. Therefore, when pig cadavers are used to inventory local carrion-arthropod faunas, they appear to be as good as humans and are more practical (Table 3).

However, we suggest that pig cadavers larger than the recommended 20–30 kg domestic pigs [9, 10] should be used to compile full inventories of carrion entomofauna because smaller pigs yield an incomplete insect inventory (i.e. underrepresentation of middle or late colonizers [44, 45]). We therefore recommend cadavers a starting mass of at least 40 kg (and preferably 50–80 kg) as a standard to investigate local carrion-insect inventories. Smaller cadavers (piglets or rabbits) may be used in cases when early colonizers (e.g. blow flies) are the focus.

Most methods developed in forensic entomology or taphonomy are intended to be used with human cadavers. Therefore, at least their final validation should be performed with humans and preferably in real case scenarios. We are not aware, however, of any validation experiment in which performance of the forensic method developed using non-human cadavers has been evaluated using human cadavers. This is definitely an area for future experiments. Such research could enable forensic scientists to evaluate whether techniques based on data from human analogues (e.g. pig cadavers) are satisfactorily accurate when used in casework for human cadavers. As a result, we could distinguish techniques for which reference data could be amassed using human cadaver analogues and techniques for which human cadavers are necessary to get reference data. Nevertheless, analogues for humans, particularly large-bodied species, serve well in “proof-of-concept” studies (Table 2). Similarly, initial validation of forensic methods may be efficiently performed with pig cadavers (Table 2), particularly when different cadaver traits (e.g. mass) or environmental conditions (e.g. below/above ground) are to be compared.

All animals used in forensic entomology or taphonomy research are highly variable within species. This may lead to misinterpretation of experimental results, particularly when the experimental design of a study has weaknesses (see section 4 of this paper). However, the variation may also be advantageous, as it enables the researcher to choose the model best suited to the research. For example, if the scientific question obliges large replication, the experiment simply cannot be made with large pigs within standard research budgets, whereas piglets may be appropriate. If the researcher is interested in the thermal profile of decomposing remains, it may be more important to focus on the sunlight absorbance and mass of the model species than on its other traits. This argument may be extended to different animal models: experiments on initial colonisation patterns of blow flies may be more tractable using piglets or rabbits rather than adult pig or human cadavers. On the other hand, validation of the total body score (TBS) method for PMI estimation [51] needs humans or at least large pigs. Therefore, there is no universal model cadaver for research in forensic taphonomy or entomology, and the one that should be chosen depends on the scientific question and its experimental demands. This is an important point for the forensic science community to consider when designing experiments, analysing results or extrapolating conclusions.

## Critique of the pig model as an analogue for human cadavers

### Background

Use of domestic pigs in experimental forensic sciences has been challenged by recent comparisons of pig and human cadaver decomposition [23, 24]. One study [23] concluded that “pigs are not an adequate proxy for human decomposition studies”, and another [24] indicated that neither rabbits nor pigs “captured the pattern, rate, and variability of human decomposition”. Pigs may indeed decompose differently to humans, and therefore their experimental comparison is clearly worthwhile to forensic sciences. However, the intrinsic logistical difficulties associated with experiments involving human cadavers may impair such comparisons (Table 3), and therefore, questions arise about the validity of recent findings and conclusions. In the following sections, we discuss these questions and try to identify their consequences for the findings of the referenced experiments [23, 24] and the implications they have for the validity of the conclusion that pigs are inadequate analogues for humans in forensic research.

**Fig. 2:**
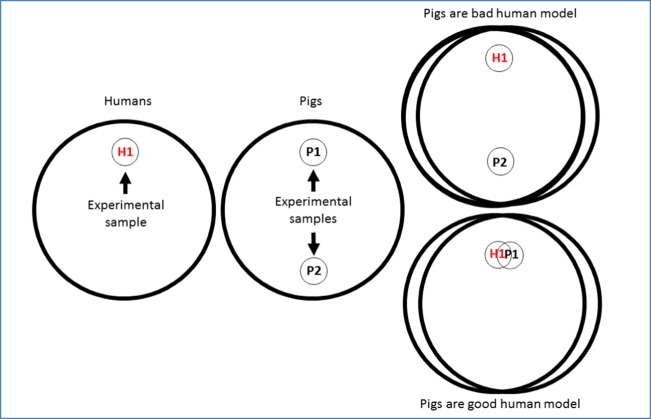
Schematic representation of dangers for human/pig comparisons, resulting from intraspecific variation of pigs and humans. Large circles are phenotype spaces (for a species), small circles inside are experimental samples of pigs or humans. The samples can come from anywhere within the phenotype space for the species, but if comparisons are to be made between species, it is desirable that the samples come from the phenotype space shared by both species. Thus, it is possible to design an experiment comparing the same two species and either properly (bottom circles, e.g. large humans versus large pigs) or improperly (upper circles, e.g. large humans versus small pigs) compare the species, depending on the choice or availability of sampled individuals

As we have discussed in section 3, all model organisms are highly variable intra-specifically. Biased sampling of this variation may lead to the misinterpretation of results of any model comparison. Both pigs and humans clearly exhibit variable sizes, pigmentation, hairiness, body mass index and other characteristics. Such factors may be confounded with treatments, and when they affect decomposition, they may make it impossible to assign results of a comparison between human and pig cadavers (or any other model) to a species effect (Fig. 2). As an example, if a study was conducted with male piglets and adult female humans only, it would not be possible to disentangle sex and age (or mass) effects from species effects. Therefore, sample selection within and between species is critical for such comparisons.

### Experimental design

#### Confounded variables

Confounded variables make the outcome of an experiment ambiguous. Confounding effects arise when differences recorded in a response (dependent) variable as a putative result of experimental manipulation of explanatory (independent) variable(s) cannot be separated from other variables that may affect the response [52]. To confidently show that differences resulted from experimental manipulations, the groups under comparison should differ only in the manipulated variable(s), or more realistically, the groups should not differ systematically in any important variable other than the one under manipulation. Confounding variables should be controlled in the experimental design (and thus eliminated) or in its statistical analysis (and thus quantified). An important confounding variable likely to arise in pig and human comparisons is body mass.

**Fig. 3:**
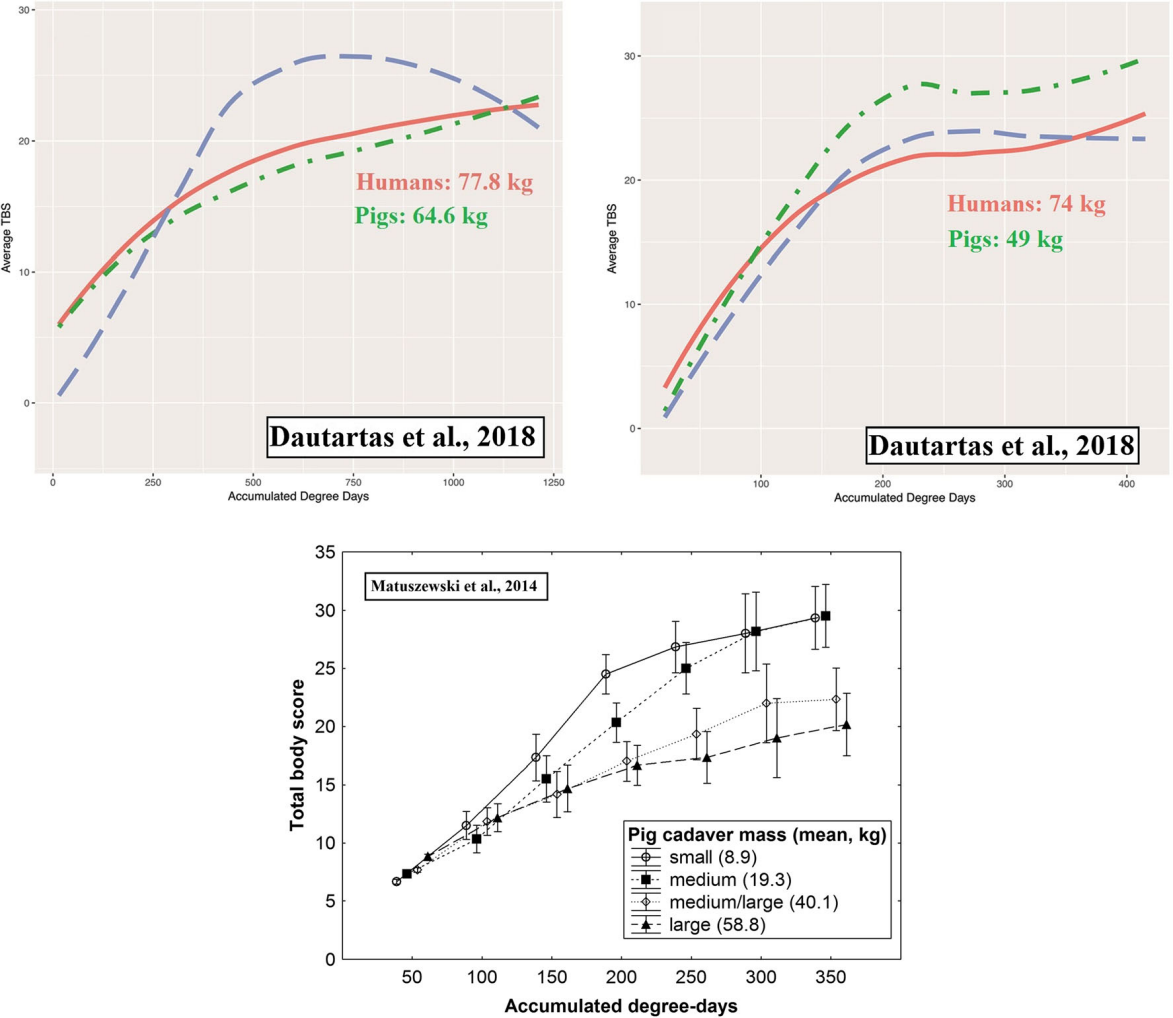
Changes in total body score (TBS) during decomposition of pig and human cadavers. Upper panel shows Fig. 2A and 2B from Dautartas et al. [24] displaying results of their trial 1 (Fig. 2A, spring, insects present) and trial 2 (figure 2B, summer, insects present). Lower figure is a modification of Fig. 13 from Matuszewski et al. [59], displaying results of their experiment with pig cadavers of different mass. Red lines in Dautartas et al. [24] are for human cadavers, green lines for pig cadavers. Comparison of the trials 1 and 2 (upper panel) indicates that an increase of difference in cadaver mass between pigs and humans in the trial 2 was followed by larger difference between TBS curves. Moreover, differences between TBS curves in the trial 2 are similar to differences between medium/large and large pig cadavers in the experiment of Matuszewski et al. [59]. Therefore, the differences between pigs and humans in Fig. 2B of Dautartas et al. [24] may be interpreted as the result of differences in mass between the cadavers and not differences in the species of cadaver

**Fig. 4:**
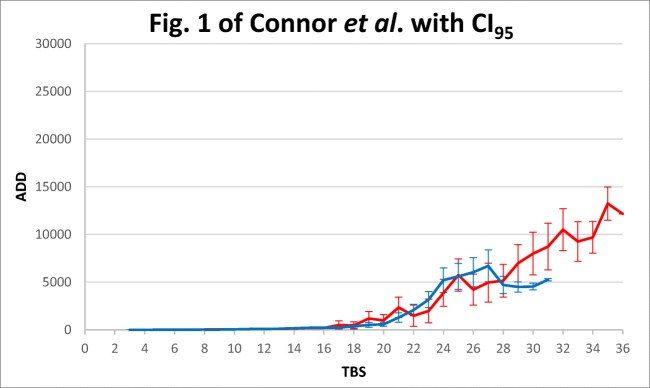
Total body score (TBS) and accumulated degree-days (ADD) with 95% confidence intervals for ADD added and plotted based on data from Table 1 of Connor et al., [23], presented by these authors (as Fig. 1) without confidence intervals. The 95% confidence intervals presented in this figure used standard deviations calculated from coefficients of variation reported in Table 1 of Connor et al. [23]. Red lines—pig cadavers; blue lines—human cadavers

Identifying differences in decomposition between species needs an experiment in which cadaver samples differ systematically only in the cadavers’ species. In the experiments of Dautartas et al. [24] and Connor et al. [23], samples of pig and human cadavers differed systematically in cadaver mass: the humans were systematically much larger than the pigs (Table 4). Although there are anecdotal observations suggesting low importance of adult human cadaver mass [53] and experimental findings supporting the claim that in a mass range of 73–159 kg (*N* = 12, nine cadavers over 100 kg, i.e. obese, adipose bodies) decomposition rate is not significantly related to human body mass [54], all rigorous studies revealed that in a forensically relevant mass range (10–90 kg) small pig cadavers decompose significantly faster than large ones [55–59]. This difference appeared only in the case of insect-colonized carcasses [56] and has been suggested to result from less efficient active decay in larger cadavers, as a consequence of competition over carrion between different insect taxa [45, 59]. It is also related to surface-to-volume ratios, which reflect the surface area of the tissue where insects can feed, and to the size of the individual insect relative to that of the resource. Based on these patterns, it may be assumed that, when insects are present, smaller pig cadavers’ progress through the TBS scale at a faster rate than larger human cadavers. This seems to be the case (Figs. 3 and 4) with the studies of Dautartas et al. [24] and Connor et al. [23], making some of their results ambiguous and uninterpretable with respect to human–pig differences.

**Table 4:** Cadaver mass of pigs and humans used by Dautartas et al. [24] and Connor et al. [23]

		Cadaver mass (kg)							
		Pigs			Difference between humans and pigs in mean cadaver mass	Humans			
		Mean	Range	V		Mean	Range	V	Dissimilarity score (h−p)/(h+p)
Dautartas et al. [24]	Trial 1	64.6	60–68	4.8	13.2	77.8	72–84	6.1	0.093
	Trial 2	49	40–59	14.1	25	74	53–107	30.8	0.203
	Trial 3	50.6	47–57	8.5	24.8	75.4	57–85	15.1	0.197
Connor et al. [23]		35 (median)	25–64	n/a	≥ 45* (median)	n/a (**≥** 80)*	n/a	n/a	0.391*

*n/a* not available

*Authors did not report mass of their human cadavers. They used adult humans and mention that “...over half the human sample was overweight or obese.”. According to “Anthropometric Reference Data for Children and Adults: United States, 2011-2014” [Fryar et al., 2016, Vital Health Stat 3] average body weight of adult females in USA was 76.4 kg and adult males 88.8 kg. Based on these data, we assume that the median mass of the human sample from Connor et al. [23] was no less than 80 kg, so the difference in median between pig and human sample was no less than 45 kg

#### Independence of replicates. Distance between cadavers

When cadavers are close to one other, they may cross-contaminate one another or “compete” for insect colonizers, or both, making them statistically non-independent [60, 61]. The cadaver that is more attractive to insects may mask the other, resulting in underrepresentation of insects and slower decomposition of the less attractive cadaver. In addition, dispersal of larvae becomes a potential mechanism to affect larval competition if the carcasses are located close to one another. If such effects are not taken into account (i.e., watching for larval dispersal, deploying drift fencing), small inter-cadaver distances are likely to alter species composition or decomposition rate, and lead to a lack of independence of experimental units, a basic assumption or requirement of most statistical tests.

In forensic entomology experiments, cadavers have usually been placed at least 50 m apart (Table 1) because there is empirical support that such a distance is sufficient to minimize cross-contamination by dispersing fly larvae [62, 63] and to ensure independence of cadavers [60]. In forensic taphonomy experiments, particularly with human cadavers, the distance has usually been much smaller, probably as a result of the smaller areas of human taphonomy research facilities where such experiments are located. Dautartas et al. [24] report that their cadavers were placed at least 3 m apart, and although Connor et al. [23] provide no information on the distance between their cadavers, the outdoor facility where the study was located has an area of about one acre [22], so we can assume their between-cadaver distances were less than 50 m. Such distances indicate that the cadavers used in both studies were not demonstrably independent in terms of the insect communities attending them. Little is known about the effect of small distance between cadavers on the pattern and rate of insect-mediated decomposition [60, 61]; therefore, relevant consequences of small between-cadaver distance on the results of the above studies are currently difficult to identify.

#### Inter-annual effects

Different years generally have different weather profiles leading to different insect richness and abundances and/or different insect pre-appearance intervals (PAI) (Table 1). These may result in substantial annual differences in decomposition rate.

In the experiments of Connor et al. [23], pig cadavers were exposed in September 2012 through August 2013 (12 pigs, one each month), while an extra five pigs were exposed on the same day as their 2nd through 6th human cadaver. The authors gave no specific dates of the human cadaver exposure (between September 2012 and December 2015). However, according to Wikipedia [22], they started to use human cadavers at their outdoor facility in November 2013. Therefore, most pigs were exposed in 2012 and 2013 and most humans probably in 2014 and 2015. If that was the case, there was a high level of treatment segregation and the species effect was confounded with an inter-annual effect. Consequently, the findings reported by Connor et al. [23] may be the result, at least in part, of differences in the biotic and abiotic determinants of decomposition in the different years of exposure rather than differences between cadaver species.

#### Subject variables

Subject variables are characteristics of individuals that are idiosyncratic and may affect the research variables, primarily by increasing their measured variances, sometimes referred to as “statistical noise”. Wherever possible, such variables should be controlled by selecting experimental subjects to minimize their effects, usually through matching the individuals as closely as possible. This is generally possible with pigs or rabbits but can be impractical with humans. For instance, the study of Connor et al. [23] exposed some human cadavers effectively fresh at the day of death but others after 53 days of postmortem refrigeration. Refrigeration affects bacterial communities that initiate decomposition, with consequences for the rate of decomposition and the attraction of insects [64], which must have resulted in amplifying variation in decomposition rates of humans in that study. This sort of consequence of working with human cadavers may predispose a study to generate misleading results.

#### Quantifying decomposition

The total body score (TBS) was originally developed as a point-based, semi-quantitative scale for scoring the decomposition of human cadavers [51]. It represents the total amount of accumulated decomposition identified from three body regions (head and neck, trunk, and limbs). The scale was modified for rabbit [25] and pig cadavers [65]. Keough et al. [65] observed significant differences between pig and human cadavers during early decomposition and proposed the amendment of the TBS scale for pig cadavers. The use of the same TBS scale to compare human and pig decomposition rate (e.g. [23, 24]) is incorrect. Given the differences observed between human and pig cadavers in gross morphological changes during decomposition [23, 24, 65], cross-species use of the same TBS scale is risky and should, ideally, be complemented with other measures of decomposition, such as daily or periodic weight loss (in %).

### Statistical analysis and the presentation of results

Criticism is essential to the advancement of science but for a critique to be acceptable, its analysis must be robust. However, the analyses presented in Connor et al. [23] and Dautartas et al. [24] are inadequate to support their conclusions. In Connor et al. [23], the conclusion of a difference between human and pig cadavers is derived from a comparison of the slopes developed using linear mixed modelling. However, a simple look at the regression lines used to compare decomposition rates (see Fig. 4 in Connor et al. [23]) shows that the selected models are inadequate in terms of adjustment, leverage values and residuals. The figure also demonstrates that a statistical difference is found by the authors only because pigs were allowed to decompose for a longer period, as no human cadaver was scored at TBS values > 31. TBS values > 31 had a powerful leverage effect on the regression line because these scores were squared in the analysis. The analyses of Dautartas et al. [24] are also problematic because none of them accounts for repeated measurements on cadavers, resulting in temporal pseudoreplication, which is known to artificially decrease *P* values.

In addition, statistically detectable effects may be too small or too variable to have practical significance for estimates of PMIs [66]. Because cadavers are highly variable, not surprisingly, decomposition rates can be highly variable too. For this reason, when trends are reported, they should be accompanied by quantitative indications of variation (i.e. uncertainty). For instance, human and pig cadavers appeared to decompose differently in the study of Connor et al. [23], but when 95% confidence intervals are added to the trend lines (Fig. 4), the apparent differences disappear. The inclusion of those intervals would indicate that pigs of small size are adequate models for human decomposition unless the TBS is greater than 28, which is a different interpretation from the one originally drawn from that research.

## Alternative model organisms

In some countries, pigs are not a realistic option for religious reasons, and other animal models are needed. Rabbits have been frequently used by forensic entomologists (Table 1), but obviously, they are too small to serve well for most forensic research. Carrion insect assemblages are distinctly less complex and persist for less time on small-sized cadavers compared with larger cadavers [44, 45]. Owing to their small size, the decomposition rate of rabbit cadavers is much faster than that of pig or human cadavers [24, 44]. Accordingly, the well-established importance of body size needs to be remembered when selecting alternatives, like sheep or goats, usually shorn to make insect sampling feasible and to reduce the potential impact of the fleece on decomposition, which is different from pig and human situations.

## Recommendations

Previous papers suggested that a universal model cadaver for experimental field studies and training programs in forensic entomology would be a domestic pig weighing 20–30 kg of starting mass [9, 10]. No recommendation is currently available for taphonomy studies. However, a single and universal “model cadaver” for the forensic sciences is not useful. Different studies have different purposes, conditions and limitations. Therefore, more flexible guidelines on cadaver species and mass are needed (Table 5). A review of the guidelines proposed in this paper (Table 5) indicates that human cadavers appear necessary only in comparative studies involving other cadaver taxa and for final validation of forensic methods. In most cases, pig cadavers are an ideal choice, whereas other animal cadavers may be useful in supplemental or unavoidable (substitutional) cases. Moreover, researchers should usually use cadavers that are larger than the currently recommended size of 20–30 kg. Depending on the specific question of interest, other non-mass-related considerations may also be necessary.

**Table 5:** Guidelines for cadaver choice in forensic science research

Research type/subtype		Guidelines	
		Cadaver species	Cadaver mass
Experimental studies		Domestic pig, rabbit or rodents, depending on the objective of the study, human for model comparison experiments	Depending on study objective
Local insect inventory or succession studies, insect PAI studies	Early colonizers	Domestic pig, rabbit	No cadaver mass limitations
	Early and middle colonizers	Domestic pig	**≥** 20 kg starting mass, preferably 20–40 kg
	All colonizers	Domestic pig	**≥** 40 kg starting mass, preferably 50–80 kg
Tests of forensic methods	Proof-of-concept studies	Domestic pig, rabbit or rodents, depending on method tested	Depending on method tested
	Initial validation studies	Domestic pig	10–40 kg as juvenile analogues, 50–80 kg as adult analogues
	Final validation studies	Human	Preferably whole mass range

## Conclusions

Pig cadavers have provided a comprehensive experimental foundation for empirical studies of decomposition in forensic entomology, taphonomy and ecology, and are likely to remain the analogue of choice in most such studies for the immediate future. A pivotal limitation to the value of human cadavers is an adequate supply of donated bodies, especially when a well-replicated experiment is required. Some of these limitations can be avoided by conducting observational studies with samples derived from death investigations (i.e. through collaboration with medical examiners), which will be limited by the samples available, and may not be appropriate for all types of scientific questions. Analogue models such as pigs are likely to remain logistically more tractable, being more readily available, more uniform in size and age and less ethically complex to deploy. Pigs are a sensible compromise between availability, cost, ethics and similarity to humans, and there is no better candidate at this time. At present, experiments using analogues are easier to replicate and make control of confounding factors more practicable than studies based solely on humans, and they can be validated by including human remains alongside the analogues (e.g. [14, 44]). Therefore, an adequate query is not whether we should abandon pig carcasses, but rather how pig carcasses and other animal models differ from human cadavers in certain aspects of their decomposition, for example, decomposition rate and patterns of colonisation by insects. Such research would put into perspective all the developments made possible over the past four decades by the use of human analogues (Table 1). Moreover, human cadavers are definitely limited resources for forensic sciences. Therefore, they should be invested to test hypotheses which were found to be forensically interesting for analogues, e.g. pig carcasses.

The need for robust replication and control are a direct consequence of both the inherent complexity of animal decomposition and the need for reliable forensic evidence in court. Our recommendations provide a quality assurance baseline for cadaver experiments. Indeed, simulated and reconstructed casework using pigs is an ideal test and cross-validation of conditions at a death scene (i.e. litigation research). Pig carcasses should be placed, if possible and acceptable, at or near the same site and time of year as the death scene and should serve as a reference for case analyses [67, 68].

A certain level of imprecision is inevitable even in superbly designed decomposition experiments, and court testimony will always need to draw cross-validation of decomposition-based estimates from other fields of science. Future decomposition studies will need to underpin their own importance with rigorous quality control measures [27, 28]. A means to this end have been outlined here, and many of the recommendations apply as much to research with human corpses as to any other animal species.

## Electronic supplementary material

ESM 1 (DOCX 50 kb)
