# Supplementary material for: Pigs vs people: the use of pigs as analogues for humans in forensic entomology and taphonomy research
Source: Int J Legal Med. 2019 Jun 17;134(2):793–810. doi: 10.1007/s00414-019-02074-5 (PMC7044136; doi:10.1007/s00414-019-02074-5)
Supplement: Supplementary file 1 — (DOCX 50 kb) [file 414_2019_2074_MOESM1_ESM.docx]

SUPPLEMENTARY REFERENCES FOR

“Pigs vs people: the use of pigs as analogues for humans in forensic entomology and taphonomy research”

International Journal of Legal Medicine

Content

1. References to Table 1 of the main text
2. References to Table 2 of the main text
3. References to Table 1

1. Chapman R, Sankey J (1955) The larger invertebrate fauna of three rabbit carcasses. Journal of Animal Ecology 24 (2):395-402

2. Bornemissza G (1957) An analysis of Arthropod succession in Carrion and the effect of its decomposiion on the soil fauna. Australian Journal of Zoology 5 (1):1-12

3. Reed HB (1958) A Study of Dog Carcass Communities in Tennessee, with Special Reference to the Insects. The American Midland Naturalist 59 (1):213-245

4. Payne JA (1965) A Summer Carrion Study of the Baby Pig Sus Scrofa Linnaeus. Ecology 46 (5):592-602

5. Payne JA, King EW, Beinhart G (1968) Arthropod succession and decomposition of buried pigs. Nature 219 (5159):1180-1181

6. Payne JAK, E.W. (1972) Insect succession and decomposition of pig carcasses in water. Journal of Georgia Entomological Society 7 (3):153-162

7. Nabagło L (1973) Participation of invertebrates in decomposition of rodent carcasses in forest ecosystems. Ekologia Polska 21:251-270

8. Cornaby BW (1974) Carrion Reduction by Animals in Contrasting Tropical Habitats. Biotropica 6 (1):51-63. doi:10.2307/2989697

9. Johnson MD (1975) Seasonal and microseral variations in the insect populations on carrion. American Midland Naturalist:79-90

10. Smith KG (1975) The faunal succession of insects and other invertebrates on a dead fox. Entomological Gazette 26:277

11. Coe M (1978) The decomposition of elephant carcasses in the Tsavo (East) National Park, Kenya. Journal of Arid Environments 1:71-86

12. McKinnerney M (1978) Carrion communities in the northern Chihuahuan Desert. The Southwestern Naturalist:563-576

13. Jiron LF, Cartin VM (1981) Insect Succession in the Decomposition of a Mammal in Costa Rica. Journal of the New York Entomological Society 89 (3):158-165

14. Abell D, Wasti S, Hartmann G (1982) Saprophagous arthropod fauna associated with turtle carrion. Applied Entomology and Zoology 17 (3):301-307

15. Rodriguez WC, Bass, W. M. (1983) Insect activity and its relationship to decay rates of human cadavers in east Tennessee. Journal of Forensic Sciences 28:423-432

16. Schoenly K, Reid, W. (1983) Community structure of carrion arthropods in the Chihuahuan Desert. Journal of Arid Environments 6:253-263

17. Lord WD, Burger JF (1984) Arthropods associated with herring gull (Larus argentatus) and great black-backed gull (Larus marinus) carrion on islands in the Gulf of Maine. Environmental Entomology 13 (5):1261-1268

18. Rodriguez WC, Bass WM (1985) Decomposition of buried bodies and methods that may aid in their location. Journal of Forensic Sciences 30 (3):836-852

19. Early M, Goff ML (1986) Arthropod succession patterns in exposed carrion on the island of O'ahu, Hawaiian Islands, USA. Journal of Medical Entomology 23 (5):520-531

20. Micozzi M (1986) Experimental study of postmortem change under field conditions : effects of freezing, thawing and mechanical injury. Journal of Forensic Sciences 31 (3):953-961

21. Braack LEO (1987) Community dynamics of carrion-attendant arthropods in tropical african woodland. Oecologia 72 (3):402-409

22. Peschke K, Krapf D, Fuldner D (1987) Ecological separation, functional relationships, and limiting resources in a carrion insect community. Zoologishe Jahrbücher Abteilung für Systematik, Ökologie und Geographie der Tiere 114:241-265

23. Tullis K, Goff ML (1987) Arthropod succession in exposed carrion in a tropical rainforest on O'ahu Island, Hawai'i. Journal of Medical Entomology 24 (3):332-339

24. Blackith RE, Blackith RM (1990) Insect infestations of small corpses. Journal of Natural History 24 (3):699-709

25. Kentner E, Streit, B. (1990) Temporal distribution and habitat preference of congeneric insect species found at rat carrion. Pedobiologia 34:347-359

26. Hewadikaram KA, Goff ML (1991) Effect of carcass size on rate of decomposition and arthropod succession patterns. The American Journal of Forensic Medicine and Pathology 12 (3):235-240

27. Vass AA, Bass WM, Wolt JD, Foss JE, Ammons JT (1992) Time since death determinations of human cadavers using soil solution. Journal of Forensic Sciences 37 (5):1236-1253

28. Shean BS, Messinger L, Papworth M (1993) Observations of differential decomposition on sun exposed v. shaded pig carrion in coastal Washington State. Journal of Forensic Sciences 38 (4):938-949

29. Anderson GS, VanLaerhoven SL (1996) Initial studies on insect succession on carrion in southwestern British Columbia. Journal of Forensic Sciences 41:617-625

30. Tantawi TI, El-Kady EM, Greenberg B, El-Ghaffar HA (1996) Arthropod succession on exposed rabbit carrion in Alexandria, Egypt. Journal of Medical Entomology 33 (4):566-580

31. Keiper JB, Chapman EG, Foote BA (1997) Midge larvae (Diptera: Chironomidae) as indicators of postmortem submersion interval of carcasses in a woodland stream: a preliminary report. Journal of Forensic Sciences 42 (6):1074-1079

32. Richards EN, Goff ML (1997) Arthropod succession on exposed carrion in three contrasting tropical habitats on Hawaii Island, Hawaii. Journal of Medical Entomology 34 (3):328-339

33. Avila FW, Goff ML (1998) Arthropod succession patterns onto burnt carrion in two contrasting habitats in the Hawaiian Islands. Journal of Forensic Sciences 43 (3):581-586

34. Komar D, Beattie O (1998) Effects of Carcass Size on Decay Rates of Shade and Sun Exposed Carrion. Canadian Society of Forensic Science Journal 31 (1):35-43

35. Komar D, Beattie O (1998) Postmortem insect activity may mimic perimortem sexual assault clothing patterns. Journal of Forensic Sciences 43 (4):792-796

36. Tomberlin JK, Adler PH (1998) Seasonal colonization and decomposition of rat carrion in water and on land in an open field in South Carolina. Journal of Medical Entomology 35 (5):704-709

37. Bourel B, Martin-Bouyer L, Hedouin V, Cailliez JC, Derout D, Gosset D (1999) Necrophilous insect succession on rabbit carrion in sand dune habitats in northern France. Journal of Medical Entomology 36 (4):420-425

38. De Jong GD, Chadwick JW (1999) Decomposition and arthropod succession on exposed rabbit carrion during summer at high altitudes in Colorado, USA. Journal of Medical Entomology 36 (6):833-845

39. Turner B, Wiltshire P (1999) Experimental validation of forensic evidence: a study of the decomposition of buried pigs in a heavy clay soil. Forensic Science International 101 (2):113-122

40. VanLaerhoven SL, Anderson GS (1999) Insect succession on buried carrion in two biogeoclimatic zones of British Columbia. Journal of Forensic Sciences 44 (1):32-43

41. Carvalho LMLd, Thyssen PJ, Linhares AX, Palhares FAB (2000) A checklist of arthropods associated with pig carrion and human corpses in Southeastern Brazil. Memórias do Instituto Oswaldo Cruz 95 (1):135-138

42. Davis JB, Goff ML (2000) Decomposition patterns in terrestrial and intertidal habitats on Oahu Island and Coconut Island, Hawaii. Journal of Forensic Sciences 45 (4):836-842

43. Shalaby OA, deCarvalho LM, Goff ML (2000) Comparison of patterns of decomposition in a hanging carcass and a carcass in contact with soil in a xerophytic habitat on the Island of Oahu, Hawaii. Journal of Forensic Sciences 45 (6):1267-1273

44. Arnaldos I, Romera E, Garcia MD, Luna A (2001) An initial study on the succession of sarcosaprophagous Diptera (Insecta) on carrion in the southeastern Iberian peninsula. International Journal of Legal Medicine 114 (3):156-162

45. Carvalho LML, Linhares AX (2001) Seasonality of insect succession and pig carcass decomposition in a natural forest area in southeastern Brazil. Journal of Forensic Sciences 46:604-608

46. Marchenko MI (2001) Medicolegal relevance of cadaver entomofauna for the determination of the time of death. Forensic Science International 120 (1-2):89-109

47. Wolff M, Uribe A, Ortiz A, Duque P (2001) A preliminary study of forensic entomology in Medellı́n, Colombia. Forensic Science International 120 (1–2):53-59

48. Yan F, McNally R, Kontanis EJ, Sadik OA (2001) Preliminary quantitative investigation of postmortem adipocere formation. Journal of Forensic Sciences 46 (3):609-614

49. Centeno N, Maldonado M, Oliva A (2002) Seasonal patterns of arthropods occurring on sheltered and unsheltered pig carcasses in Buenos Aires Province (Argentina). Forensic Science International 126 (1):63-70

50. Hobischak NR, Anderson GS (2002) Time of submergence using aquatic invertebrate succession and decompositional changes. Journal of Forensic Sciences 47 (1):142-151

51. Leblanc H, Strongman D (2002) Carrion insects associated with small pig carcasses during fall in Nova Scotia. Canadian Society of Forensic Science Journal 35 (3):145-152

52. Archer MS, Elgar MA (2003) Yearly activity patterns in southern Victoria (Australia) of seasonally active carrion insects. Forensic Science International 132 (3):173-176

53. Archer MS, Elgar MA (2003) Effects of decomposition on carcass attendance in a guild of carrion-breeding flies. Medical and Veterinary Entomology 17 (3):263-271

54. Bharti M, Singh D (2003) Insect faunal succession on decaying rabbit carcasses in Punjab, India. Journal of Forensic Sciences 48 (5):1133-1143

55. Kočárek P (2003) Decomposition and Coleoptera succession on exposed carrion of small mammal in Opava, the Czech Republic. European Journal of Soil Biology 39 (1):31-45

56. Shahid SA, Schoenly K, Haskell NH, Hall RD, Zhang W (2003) Carcass enrichment does not alter decay rates or arthropod community structure: a test of the arthropod saturation hypothesis at the anthropology research facility in Knoxville, Tennessee. Journal of Medical Entomology 40 (4):559-569

57. Watson E, Carlton C (2005) Succession of forensically significant carrion beetle larvae on large carcasses (Coleoptera: Silphidae). Southeastern Naturalist 4 (2):335-346

58. Watson EJ, Carlton CE (2005) Insect succession and decomposition of wildlife carcasses during fall and winter in Louisiana. Journal of Medical Entomology 42 (2):193-203

59. Watson EJ, Carlton CE (2003) Spring succession of necrophilous insects on wildlife carcasses in Louisiana. Journal of Medical Entomology 40 (3):338-347

60. Anderson GS, Hobischak NR (2004) Decomposition of carrion in the marine environment in British Columbia, Canada. International Journal of Legal Medicine 118 (4):206-209

61. Archer MS (2004) Rainfall and temperature effects on the decomposition rate of exposed neonatal remains. Science & Justice 44 (1):35-41

62. Archer MS (2004) Annual variation in arrival and departure times of carrion insects at carcasses: implications for succession studies in forensic entomology. Australian Journal of Zoology 51 (6):569-576

63. Arnaldos MI, Romera E, Presa JJ, Luna A, Garcia MD (2004) Studies on seasonal arthropod succession on carrion in the southeastern Iberian Peninsula. International Journal of Legal Medicine 118 (4):197-205

64. Grassberger M, Frank C (2004) Initial study of arthropod succession on pig carrion in a central European urban habitat. Journal of Medical Entomology 41 (3):511-523

65. Tabor KL, Fell RD, Brewster CC (2005) Insect fauna visiting carrion in Southwest Virginia. Forensic Science International 150 (1):73-80

66. Tabor KL, Brewster CC, Fell RD (2004) Analysis of the successional patterns of insects on carrion in southwest Virginia. Journal of Medical Entomology 41 (4):785-795

67. Vass AA, Smith RR, Thompson CV, Burnett MN, Wolf DA, Synstelien JA, Dulgerian N, Eckenrode BA (2004) Decompositional odor analysis database. Journal of Forensic Sciences 49 (4):760-769

68. Anderson GS (2005) Effects of Arson on Forensic Entomology Evidence. Canadian Society of Forensic Science Journal 38 (2):49-67

69. Moura MO, Monteiro-Filho ELdA, Carvalho CJBd (2005) Heterotrophic succession in carrion arthropod assemblages. Brazilian Archives of Biology and Technology 48:477-486

70. Perez SP, Duque P, Wolff M (2005) Successional behavior and occurrence matrix of carrion-associated arthropods in the urban area of Medellin, Colombia. Journal of Forensic Sciences 50 (2):448-454

71. Schoenly KG, Shahid SA, Haskell NH, Hall RD (2005) Does carcass enrichment alter community structure of predaceous and parasitic arthropods? A second test of the arthropod saturation hypothesis at the Anthropology Research Facility in Knoxville, Tennessee. Journal of Forensic Sciences 50 (1):134-142

72. Weitzel MA (2005) A report of decomposition rates of a special burial type in Edmonton, Alberta from an experimental field study. Journal of Forensic Sciences 50 (3):641-647

73. De Jong GD, Hoback WW (2006) Effect of investigator disturbance in experimental forensic entomology: succession and community composition. Medical and Veterinary Entomology 20 (2):248-258

74. De Jong GD, Hoback WW, Higley LG (2011) Effect of Investigator Disturbance in Experimental Forensic Entomology: Carcass Biomass Loss and Temperature. Journal of Forensic Sciences 56 (1):143-149

75. Hobischak NR, VanLaerhoven SL, Anderson GS (2006) Successional patterns of diversity in insect fauna on carrion in sun and shade in the Boreal Forest Region of Canada, near Edmonton, Alberta. Canadian Entomologist 138 (3):376-383

76. Joy JE, Liette NL, Harrah HL (2006) Carrion fly (Diptera: Calliphoridae) larval colonization of sunlit and shaded pig carcasses in West Virginia, USA. Forensic Science International 164 (2-3):183-192

77. Lang MD, Allen GR, Horton BJ (2006) Blowfly succession from possum (Trichosurus vulpecula) carrion in a sheep-farming zone. Medical and Veterinary Entomology 20 (4):445-452

78. Adlam RE, Simmons T (2007) The effect of repeated physical disturbance on soft tissue decomposition--are taphonomic studies an accurate reflection of decomposition? Journal of Forensic Sciences 52 (5):1007-1014

79. Gruner SV, Slone DH, Capinera JL (2007) Forensically important calliphoridae (diptera) associated with pig carrion in rural north-central Florida. Journal of Medical Entomology 44 (3):509-515

80. Martinez E, Duque P, Wolff M (2007) Succession pattern of carrion-feeding insects in Paramo, Colombia. Forensic Science International 166 (2-3):182-189

81. O'Brien RC, Forbes SL, Meyer J, Dadour IR (2007) A preliminary investigation into the scavenging activity on pig carcasses in Western Australia. Forensic Science, Medicine and Pathology 3 (3):194-199

82. Schoenly KG, Haskell NH, Hall RD, Gbur JR (2007) Comparative performance and complementarity of four sampling methods and arthropod preference tests from human and porcine remains at the Forensic Anthropology Center in Knoxville, Tennessee. Journal of Medical Entomology 44 (5):881-894

83. Benninger LA, Carter DO, Forbes SL (2008) The biochemical alteration of soil beneath a decomposing carcass. Forensic Science International 180 (2-3):70-75

84. Eberhardt TL, Elliot DA (2008) A preliminary investigation of insect colonisation and succession on remains in New Zealand. Forensic Science International 176 (2-3):217-223

85. Fiedler A, Halbach M, Sinclair B, Benecke M (2008) What is the edge of a forest? A diversity analysis of adult Diptera found on decomposing piglets inside and on the edge of a western German woodland inspired by a courtroom question. Entomologie Heute 20:173-191

86. Huntington TE, Carter DO, Higley LG (2008) Testing multigenerational colonization of carrion by blow flies in the Great Plains. Great Plains Research 18 (1):33-38

87. Matuszewski S, Bajerlein D, Konwerski S, Szpila K (2008) An initial study of insect succession and carrion decomposition in various forest habitats of Central Europe. Forensic Science International 180 (2-3):61-69

88. Moretti TdC, Ribeiro OB, Thyssen PJ, Solis DR (2008) Insects on decomposing carcasses of small rodents in a secondary forest in Southeastern Brazil. European Journal of Entomology 105 (4):691-696

89. Sharanowski BJ, Walker EG, Anderson GS (2008) Insect succession and decomposition patterns on shaded and sunlit carrion in Saskatchewan in three different seasons. Forensic Science International 179 (2-3):219-240

90. Ururahy-Rodrigues A, Rafael JA, Wanderley RF, Marques H, Pujol-Luz JR (2008) Coprophanaeus lancifer (Linnaeus, 1767)(Coleoptera, Scarabaeidae) activity moves a man-size pig carcass: Relevant data for forensic taphonomy. Forensic Science International 182 (1-3):e19-e22

91. Voss SC, Forbes SL, Dadour IR (2008) Decomposition and insect succession on cadavers inside a vehicle environment. Forensic Science, Medicine and Pathology 4 (1):22-32

92. Wang J, Li Z, Chen Y, Chen Q, Yin X (2008) The succession and development of insects on pig carcasses and their significances in estimating PMI in south China. Forensic Science International 179 (1):11-18

93. Charabidze D, Bourel B, Hedouin V, Gosset D (2009) Repellent effect of some household products on fly attraction to cadavers. Forensic Science International 189 (1-3):28-33

94. Dekeirsschieter J, Verheggen FJ, Gohy M, Hubrecht F, Bourguignon L, Lognay G, Haubruge E (2009) Cadaveric volatile organic compounds released by decaying pig carcasses (Sus domesticus L.) in different biotopes. Forensic Science International 189 (1-3):46-53

95. Kalinova B, Podskalska H, Ruzicka J, Hoskovec M (2009) Irresistible bouquet of death--how are burying beetles (Coleoptera: Silphidae: Nicrophorus) attracted by carcasses. Die Naturwissenschaften 96 (8):889-899

96. Kelly JA, van der Linde TC, Anderson GS (2009) The influence of clothing and wrapping on carcass decomposition and arthropod succession during the warmer seasons in central South Africa. Journal of Forensic Sciences 54 (5):1105-1112

97. Kelly JA, van der Linde TC, Anderson GS (2011) The influence of wounds, severe trauma, and clothing, on carcass decomposition and arthropod succession in South Africa. Canadian Society of Forensic Science Journal 44 (4):144-157

98. Kjorlien YP, Beattie OB, Peterson AE (2009) Scavenging activity can produce predictable patterns in surface skeletal remains scattering: Observations and comments from two experiments. Forensic Science International 188 (1-3):103-106

99. Nelder MP, McCreadie JW, Major CS (2009) Blow flies visiting decaying alligators: is succession synchronous or asynchronous? Psyche: A Journal of Entomology 2009

100. Özdemir S, Sert O (2009) Determination of Coleoptera fauna on carcasses in Ankara province, Turkey. Forensic Science International 183 (1–3):24-32

101. Pakosh CM, Rogers TL (2009) Soft tissue decomposition of submerged, dismembered pig limbs enclosed in plastic bags. Journal of Forensic Sciences 54 (6):1223-1228

102. Parmenter RR, MacMahon JA (2009) Carrion decomposition and nutrient cycling in a semiarid shrub–steppe ecosystem. Ecological Monographs 79 (4):637-661

103. Segura NA, Usaquén W, Sánchez MC, Chuaire L, Bello F (2009) Succession pattern of cadaverous entomofauna in a semi-rural area of Bogotá, Colombia. Forensic Science International 187 (1-3):66-72

104. Van Belle LE, Carter DO, Forbes SL (2009) Measurement of ninhydrin reactive nitrogen influx into gravesoil during aboveground and belowground carcass (Sus domesticus) decomposition. Forensic Science International 193 (1-3):37-41

105. Voss SC, Spafford H, Dadour IR (2009) Annual and seasonal patterns of insect succession on decomposing remains at two locations in Western Australia. Forensic Science International 193 (1-3):26-36

106. Bachmann J, Simmons T (2010) The influence of preburial insect access on the decomposition rate. Journal of Forensic Sciences 55 (4):893-900

107. Battán Horenstein M, Linhares AX (2011) Seasonal composition and temporal succession of necrophagous and predator beetles on pig carrion in central Argentina. Medical and Veterinary Entomology 25 (4):395-401

108. Battán Horenstein M, Linhares AX, De Ferradas BR, Garcia D (2010) Decomposition and dipteran succession in pig carrion in central Argentina: ecological aspects and their importance in forensic science. Medical and Veterinary Entomology 24 (1):16-25

109. Battán Horenstein M, Rosso B, García MD (2012) Seasonal structure and dynamics of sarcosaprophagous fauna on pig carrion in a rural area of Cordoba (Argentina): Their importance in forensic science. Forensic Science International 217 (1–3):146-156

110. Bonacci T, Brandmayr P, Greco S, Tersaruolo C, Vercillo V, Brandmayr TZB (2010) A preliminary investigation of insect succession on carrion in Calabria (southern Italy). Terrestrial Arthropod Reviews 3 (2):97-110

111. Carter DO, Yellowlees D, Tibbett M (2010) Moisture can be the dominant environmental parameter governing cadaver decomposition in soil. Forensic Science International 200 (1-3):60-66

112. Chin HC, Sulaiman S, Othman H, Jeffery J, Kurahashi H, Omar B (2010) Insect succession associated with a hanging pig carcass placed in an oil palm plantation in Malaysia. Sains Malaysiana 39 (6):921-926

113. Cross P, Simmons T (2010) The influence of penetrative trauma on the rate of decomposition. Journal of Forensic Sciences 55 (2):295-301

114. Matuszewski S, Bajerlein D, Konwerski S, Szpila K (2010) Insect succession and carrion decomposition in selected forests of Central Europe. Part 2: Composition and residency patterns of carrion fauna. Forensic Science International 195 (1-3):42-51

115. Matuszewski S, Bajerlein D, Konwerski S, Szpila K (2010) Insect succession and carrion decomposition in selected forests of Central Europe. Part 1: Pattern and rate of decomposition. Forensic Science International 194 (1-3):85-93

116. Matuszewski S, Bajerlein D, Konwerski S, Szpila K (2011) Insect succession and carrion decomposition in selected forests of Central Europe. Part 3: Succession of carrion fauna. Forensic Science International 207 (1-3):150-163

117. Michaud JP, Majka CG, Prive JP, Moreau G (2010) Natural and anthropogenic changes in the insect fauna associated with carcasses in the North American Maritime lowlands. Forensic Science International 202 (1-3):64-70

118. Reibe S, Madea B (2010) How promptly do blowflies colonise fresh carcasses? A study comparing indoor with outdoor locations. Forensic Science International 195 (1-3):52-57

119. Sabanoglu B, Sert O (2010) Determination of Calliphoridae (Diptera) fauna and seasonal distribution on carrion in Ankara province. Journal of Forensic Sciences 55 (4):1003-1007

120. Simmons T, Cross PA, Adlam RE, Moffatt C (2010) The influence of insects on decomposition rate in buried and surface remains. Journal of Forensic Sciences 55 (4):889-892

121. Simmons T, Adlam RE, Moffatt C (2010) Debugging decomposition data--comparative taphonomic studies and the influence of insects and carcass size on decomposition rate. Journal of Forensic Sciences 55 (1):8-13

122. Swann L, Chidlow GE, Forbes S, Lewis SW (2010) Preliminary studies into the characterization of chemical markers of decomposition for geoforensics. Journal of Forensic Sciences 55 (2):308-314

123. Swann L, Forbes S, Lewis SW (2010) Observations of the temporal variation in chemical content of decomposition fluid: A preliminary study using pigs as a model system. Australian Journal of Forensic Sciences 42 (3):199-210

124. Szpila K, Voss JG, Pape T (2010) A new dipteran forensic indicator in buried bodies. Medical and Veterinary Entomology 24 (3):278-283

125. Valdes-Perezgasga MT, Sanchez-Ramos FJ, Garcia-Martinez O, Anderson GS (2010) Arthropods of forensic importance on pig carrion in the Coahuilan semidesert, Mexico. Journal of Forensic Sciences 55 (4):1098-1101

126. Ahmad A, Ahmad AH, Dieng H, Satho T, Ahmad H, Aziz AT, Boots M (2011) Cadaver wrapping and arrival performance of adult flies in an oil palm plantation in northern peninsular Malaysia. Journal of Medical Entomology 48 (6):1236-1246

127. Anderson GS (2011) Comparison of decomposition rates and faunal colonization of carrion in indoor and outdoor environments. Journal of Forensic Sciences 56 (1):136-142

128. Anton E, Niederegger S, Beutel RG (2011) Beetles and flies collected on pig carrion in an experimental setting in Thuringia and their forensic implications. Medical and Veterinary Entomology 25 (4):353-364

129. Barrios M, Wolff M (2011) Initial study of arthropods succession and pig carrion decomposition in two freshwater ecosystems in the Colombian Andes. Forensic Science International 212 (1-3):164-172

130. Bajerlein D, Matuszewski S, Konwerski S (2011) Insect succession on carrion: Seasonality, habitat preference and residency of histerid beetles (Coleoptera: Histeridae) visiting pig carrion exposed in various forests (Western Poland). Polish Journal of Ecology 59 (4):789-797

131. Bugajski KN, Seddon CC, Williams RE (2011) A comparison of blow fly (Diptera: Calliphoridae) and beetle (Coleoptera) activity on refrigerated only versus frozen-thawed pig carcasses in Indiana. Journal of Medical Entomology 48 (6):1231-1235

132. Cassar J, Stuart B, Dent B, Notter S, Forbes S, O'Brien C, Dadour I (2011) A study of adipocere in soil collected from a field leaching study. Australian Journal of Forensic Sciences 43 (1):3-11

133. DeVault TL, Olson ZH, Beasley JC, Rhodes Jr OE (2011) Mesopredators dominate competition for carrion in an agricultural landscape. Basic and Applied Ecology 12 (3):268-274

134. Dickson GC, Poulter RT, Maas EW, Probert PK, Kieser JA (2011) Marine bacterial succession as a potential indicator of postmortem submersion interval. Forensic Science International 209 (1-3):1-10

135. von Hoermann C, Ruther J, Reibe S, Madea B, Ayasse M (2011) The importance of carcass volatiles as attractants for the hide beetle *Dermestes maculatus* (De Geer). Forensic Science International 212 (1–3):173-179

136. Spicka A, Johnson R, Bushing J, Higley LG, Carter DO (2011) Carcass mass can influence rate of decomposition and release of ninhydrin-reactive nitrogen into gravesoil. Forensic Science International 209 (1-3):80-85

137. Statheropoulos M, Agapiou A, Zorba E, Mikedi K, Karma S, Pallis G, Eliopoulos C, Spiliopoulou C (2011) Combined chemical and optical methods for monitoring the early decay stages of surrogate human models. Forensic Science International 210 (1-3):154-163

138. Voss SC, Cook DF, Dadour IR (2011) Decomposition and insect succession of clothed and unclothed carcasses in Western Australia. Forensic Science International 211 (1-3):67-75

139. Al-Mesbah H, Moffatt C, El-Azazy OM, Majeed QA (2012) The decomposition of rabbit carcasses and associated necrophagous Diptera in Kuwait. Forensic Science International 217 (1-3):27-31

140. Brasseur C, Dekeirsschieter J, Schotsmans EM, de Koning S, Wilson AS, Haubruge E, Focant J-F (2012) Comprehensive two-dimensional gas chromatography–time-of-flight mass spectrometry for the forensic study of cadaveric volatile organic compounds released in soil by buried decaying pig carcasses. Journal of Chromatography A 1255:163-170

141. Gruenthal A, Moffatt C, Simmons T (2012) Differential decomposition patterns in charred versus un-charred remains. Journal of Forensic Sciences 57 (1):12-18

142. Martin-Vega D, Baz A (2013) Sarcosaprophagous Diptera assemblages in natural habitats in central Spain: spatial and seasonal changes in composition. Medical and Veterinary Entomology 27 (1):64-76

143. Martín-Vega D, Baz A (2012) Spatiotemporal distribution of necrophagous beetles (Coleoptera: Dermestidae, Silphidae) assemblages in natural habitats of central Spain. Annals of the Entomological Society of America 105 (1):44-53

144. Ortloff A, Pena P, Riquelme M (2012) Preliminary study of the succession pattern of necrobiont insects, colonising species and larvae on pig carcasses in Temuco (Chile) for forensic applications. Forensic Science International 222 (1-3):e36-e41

145. Prado e Castro C, García MD, Martins da Silva P, Faria e Silva I, Serrano A (2013) Coleoptera of forensic interest: A study of seasonal community composition and succession in Lisbon, Portugal. Forensic Science International 232 (1-3):73-83

146. Prado E Castro C, Serrano A, Martins Da Silva P, García MD (2012) Carrion flies of forensic interest: A study of seasonal community composition and succession in Lisbon, Portugal. Medical and Veterinary Entomology 26 (4):417-431

147. Shelomi M, Matern LM, Dinstell JM, Harris DW, Kimsey RB (2012) DEET (N,N-diethyl-meta-toluamide) induced delay of blowfly landing and oviposition rates on treated pig carrion (Sus scrofa L.). Journal of Forensic Sciences 57 (6):1507-1511

148. Stadler S, Stefanuto P-H, Brokl M, Forbes SL, Focant J-Fo (2012) Characterization of volatile organic compounds from human analogue decomposition using thermal desorption coupled to comprehensive two-dimensional gas chromatography–time-of-flight mass spectrometry. Analytical Chemistry 85 (2):998-1005

149. Widya M, Moffatt C, Simmons T (2012) The formation of early stage adipocere in submerged remains: a preliminary experimental study. Journal of Forensic Sciences 57 (2):328-333

150. Azwandi A, Nina Keterina H, Owen L, Nurizzati M, Omar B (2013) Adult carrion arthropod community in a tropical rainforest of Malaysia: analysis on three common forensic entomology animal models. Tropical Biomedicine 30 (3):481-494

151. Barton PS, Cunningham SA, Macdonald BC, McIntyre S, Lindenmayer DB, Manning AD (2013) Species traits predict assemblage dynamics at ephemeral resource patches created by carrion. PLoS ONE 8 (1):e53961

152. Benbow ME, Lewis AJ, Tomberlin JK, Pechal JL (2013) Seasonal necrophagous insect community assembly during vertebrate carrion decomposition. Journal of Medical Entomology 50 (2):440-450

153. Bygarski K, LeBlanc HN (2013) Decomposition and arthropod succession in Whitehorse, Yukon territory, Canada. Journal of Forensic Sciences 58 (2):413-418

154. Dekeirsschieter J, Frederick C, Verheggen FJ, Drugmand D, Haubruge E (2013) Diversity of forensic rive beetles (Coleoptera, Staphylinidae) associated with decaying pig carcass in a forest biotope. Journal of Forensic Sciences 58 (4):1032-1040

155. von Hoermann C, Steiger S, Müller JK, Ayasse M (2013) Too Fresh Is Unattractive! The Attraction of Newly Emerged *Nicrophorus vespilloides* Females to Odour Bouquets of Large Cadavers at Various Stages of Decomposition. PLoS ONE 8 (3):e58524

156. Hyde ER, Haarmann DP, Lynne AM, Bucheli SR, Petrosino JF (2013) The living dead: bacterial community structure of a cadaver at the onset and end of the bloat stage of decomposition. PloS ONE 8 (10):e77733

157. Johansen H, Solum M, Knudsen GK, Hagvar EB, Norli HR, Aak A (2014) Blow fly responses to semiochemicals produced by decaying carcasses. Medical and Veterinary Entomology 28 (1):26-34

158. Johnson AP, Mikac KM, Wallman JF (2013) Thermogenesis in decomposing carcasses. Forensic Science International 231 (1-3):271-277

159. Lowe A, Beresford D, Carter D, Gaspari F, O’Brien R, Stuart B, Forbes S (2013) The effect of soil texture on the degradation of textiles associated with buried bodies. Forensic Science International 231 (1-3):331-339

160. Matuszewski S, Szafalowicz M, Jarmusz M (2013) Insects colonising carcasses in open and forest habitats of Central Europe: search for indicators of corpse relocation. Forensic Science International 231 (1-3):234-239

161. Mądra A, Konwerski S, Matuszewski S (2014) Necrophilous Staphylininae (Coleoptera: Staphylinidae) as indicators of season of death and corpse relocation. Forensic Science International 242:32-37

162. Metcalf JL, Wegener Parfrey L, Gonzalez A, Lauber CL, Knights D, Ackermann G, Humphrey GC, Gebert MJ, Van Treuren W, Berg-Lyons D, Keepers K, Guo Y, Bullard J, Fierer N, Carter DO, Knight R (2013) A microbial clock provides an accurate estimate of the postmortem interval in a mouse model system. eLife 2:e01104

163. Meyer J, Anderson B, Carter DO (2013) Seasonal variation of carcass decomposition and gravesoil chemistry in a cold (Dfa) climate. Journal of Forensic Sciences 58 (5):1175-1182

164. Sutherland A, Myburgh J, Steyn M, Becker PJ (2013) The effect of body size on the rate of decomposition in a temperate region of South Africa. Forensic Science International 231 (1-3):257-262

165. von der Lühe B, Dawson LA, Mayes RW, Forbes SL, Fiedler S (2013) Investigation of sterols as potential biomarkers for the detection of pig (S. s. domesticus) decomposition fluid in soils. Forensic Science International 230 (1-3):68-73

166. Abouzied EM (2014) Insect colonization and succession on rabbit carcasses in southwestern mountains of the Kingdom of Saudi Arabia. Journal of Medical Entomology 51 (6):1168-1174

167. Anderson GS, Bell LS (2014) Deep coastal marine taphonomy: investigation into carcass decomposition in the Saanich Inlet, British Columbia using a baited camera. PLoS ONE 9 (10):e110710

168. Bhadra P, Hart A, Hall M (2014) Factors affecting accessibility to blowflies of bodies disposed in suitcases. Forensic Science International 239:62-72

169. Caballero U, León-Cortés JL (2014) Beetle succession and diversity between clothed sun-exposed and shaded pig carrion in a tropical dry forest landscape in Southern Mexico. Forensic Science International 245:143-150

170. Corrêa R, Almeida L, Moura M (2014) Coleoptera associated with buried carrion: potential forensic importance and seasonal composition. Journal of Medical Entomology 51 (5):1057-1066

171. Farwig N, Brandl R, Siemann S, Wiener F, Müller J (2014) Decomposition rate of carrion is dependent on composition not abundance of the assemblages of insect scavengers. Oecologia 175 (4):1291-1300

172. Matuszewski S, Konwerski S, Fratczak K, Szafalowicz M (2014) Effect of body mass and clothing on decomposition of pig carcasses. International Journal of Legal Medicine 128 (6):1039-1048

173. Matuszewski S, Fratczak K, Konwerski S, Bajerlein D, Szpila K, Jarmusz M, Szafalowicz M, Grzywacz A, Madra A (2016) Effect of body mass and clothing on carrion entomofauna. International Journal of Legal Medicine 130 (1):221-232

174. Mądra A, Frątczak K, Grzywacz A, Matuszewski S (2015) Long-term study of pig carrion entomofauna. Forensic Science International 252:1-10

175. Mohr RM, Tomberlin JK (2015) Development and validation of a new technique for estimating a minimum postmortem interval using adult blow fly (Diptera: Calliphoridae) carcass attendance. International Journal of Legal Medicine 129 (4):851-859

176. Mohr RM, Tomberlin JK (2014) Environmental factors affecting early carcass attendance by four species of blow flies (Diptera: Calliphoridae) in Texas. Journal of Medical Entomology 51 (3):702-708

177. Oliveira-Costa Ja, Lamego C, Couri M, Mello-Patiu C (2014) Differential Diptera succession patterns onto partially burned and unburned pig carrion in southeastern Brazil. Brazilian Journal of Biology 74 (4):870-876

178. Pechal JL, Benbow ME, Crippen TL, Tarone AM, Tomberlin JK (2014) Delayed insect access alters carrion decomposition and necrophagous insect community assembly. Ecosphere 5 (4):1-21

179. Pechal JL, Crippen TL, Benbow ME, Tarone AM, Dowd S, Tomberlin JK (2014) The potential use of bacterial community succession in forensics as described by high throughput metagenomic sequencing. International Journal of Legal Medicine 128 (1):193-205

180. Perrault KA, Rai T, Stuart BH, Forbes SL (2015) Seasonal comparison of carrion volatiles in decomposition soil using comprehensive two-dimensional gas chromatography–time of flight mass spectrometry. Analytical Methods 7 (2):690-698

181. Perrault KA, Stefanuto P-H, Stuart BH, Rai T, Focant J-F, Forbes SL (2015) Detection of decomposition volatile organic compounds in soil following removal of remains from a surface deposition site. Forensic Science, Medicine and Pathology 11 (3):376-387

182. Perrault KA, Stuart BH, Forbes SL (2014) A longitudinal study of decomposition odour in soil using sorbent tubes and solid phase microextraction. Chromatography 1 (3):120-140

183. Whitaker AP (2014) Development of blowflies (Diptera: Calliphoridae) on pig and human cadavers - Implications for forensic entomology casework. PhD Thesis, King’s College, London, UK

184. Young A, Stillman R, Smith MJ, Korstjens AH (2014) An experimental study of vertebrate scavenging behavior in a northwest European woodland context. Journal of Forensic Sciences 59 (5):1333-1342

185. Zurawski KN, Benbow ME, Miller JR, Merritt RW (2014) Examination of nocturnal blow fly (Diptera: Calliphoridae) oviposition on pig carcasses in mid-Michigan. Journal of Medical Entomology 46 (3):671-679

186. Agapiou A, Zorba E, Mikedi K, McGregor L, Spiliopoulou C, Statheropoulos M (2015) Analysis of volatile organic compounds released from the decay of surrogate human models simulating victims of collapsed buildings by thermal desorption–comprehensive two-dimensional gas chromatography–time of flight mass spectrometry. Analytica Chimica Acta 883:99-108

187. Alexander MB, Hodges TK, Bytheway J, Aitkenhead-Peterson JA (2015) Application of soil in Forensic Science: Residual odor and HRD dogs. Forensic Science International 249:304-313

188. Aubernon C, Charabidzé D, Devigne C, Delannoy Y, Gosset D (2015) Experimental study of Lucilia sericata (Diptera Calliphoridae) larval development on rat cadavers: Effects of climate and chemical contamination. Forensic Science International 253:125-130

189. Baz A, Botias C, Martin-Vega D, Cifrian B, Diaz-Aranda LM (2015) Preliminary data on carrion insects in urban (indoor and outdoor) and periurban environments in central Spain. Forensic Science International 248:41-47

190. Card A, Cross P, Moffatt C, Simmons T (2015) The Effect of Clothing on the Rate of Decomposition and Diptera Colonization on Sus scrofa Carcasses. Journal of Forensic Sciences 60 (4):979-982

191. Farrell JF, Whittington AE, Zalucki MP (2015) A review of necrophagous insects colonising human and animal cadavers in south-east Queensland, Australia. Forensic Science International 257:149-154

192. Hyde ER, Haarmann DP, Petrosino JF, Lynne AM, Bucheli SR (2015) Initial insights into bacterial succession during human decomposition. International Journal of Legal Medicine 129 (3):661-671

193. Iancu L, Carter DO, Junkins EN, Purcarea C (2015) Using bacterial and necrophagous insect dynamics for post-mortem interval estimation during cold season: Novel case study in Romania. Forensic Science International 254:106-117

194. Iancu L, Sahlean T, Purcarea C (2015) Dynamics of necrophagous insect and tissue bacteria for postmortem interval estimation during the warm season in Romania. Journal of Medical Entomology 53 (1):54-66

195. Lynch-Aird J, Moffatt C, Simmons T (2015) Decomposition Rate and Pattern in Hanging Pigs. Journal of Forensic Sciences 60 (5):1155-1163

196. Martin-Vega D, Cifrian B, Diaz-Aranda LM, Baz A (2015) Necrophilous Histerid Beetle Communities (Coleoptera: Histeridae) in Central Spain: Species Composition and Habitat Preferences. Environmental Entomology 44 (4):966-974

197. Paczkowski S, Nicke S, Ziegenhagen H, Schütz S (2015) Volatile Emission of Decomposing Pig Carcasses (S us scrofa domesticus L.) as an Indicator for the P ostmortem Interval. Journal of Forensic Sciences 60:S130-S137

198. Roberts LG, Dabbs GR (2015) A Taphonomic Study Exploring the Differences in Decomposition Rate and Manner between Frozen and Never Frozen Domestic Pigs (S us scrofa). Journal of Forensic Sciences 60 (3):588-594

199. Rysavy NM, Goff ML (2015) Preliminary observations of arthropods associated with buried carrion on Oahu. Journal of Forensic Sciences 60 (2):462-467

200. Silahuddin SA, Latif B, Kurahashi H, Walter DE, Heo CC (2015) The importance of habitat in the ecology of decomposition on rabbit carcasses in Malaysia: implications in forensic entomology. Journal of Medical Entomology 52 (1):9-23

201. Stadler S, Desaulniers J-P, Forbes SL (2015) Inter-year repeatability study of volatile organic compounds from surface decomposition of human analogues. International Journal of Legal Medicine 129 (3):641-650

202. Sukchit M, Deowanish S, Butcher BA (2015) Decomposition stages and carrion insect succession on dressed hanging pig carcasses in Nan Province, Northern Thailand. Tropical Natural History 15 (2):137–153

203. Szpila K, Mądra A, Jarmusz M, Matuszewski S (2015) Flesh flies (Diptera: Sarcophagidae) colonising large carcasses in Central Europe. Parasitology Research 114 (6):2341-2348

204. Ueland M, Nizio KD, Forbes SL, Stuart BH (2015) The interactive effect of the degradation of cotton clothing and decomposition fluid production associated with decaying remains. Forensic Science International 255:56-63

205. Zanetti NI, Visciarelli EC, Centeno ND (2015) Associational patterns of scavenger beetles to decomposition stages. Journal of Forensic Sciences 60 (4):919-927

206. Zanetti NI, Visciarelli EC, Centeno ND (2015) Trophic roles of scavenger beetles in relation to decomposition stages and seasons. Revista Brasileira de Entomologia 59 (2):132-137

207. Zeariya MG, Hammad KM, Fouda MA, Al-Dali AG, Kabadaia MM (2015) Forensic-insect succession and decomposition patterns of dog and rabbit carcasses in different habitats. Journal of Entomology and Zoology Studies 3 (5):473-482

208. Anderson GS, Bell LS (2016) Impact of Marine Submergence and Season on Faunal Colonization and Decomposition of Pig Carcasses in the Salish Sea. PLoS ONE 11 (3):e0149107

209. Cammack JA, Cohen AC, Kreitlow KL, Roe RM, Watson DW (2016) Decomposition of Concealed and Exposed Porcine Remains in the North Carolina Piedmont. Journal of Medical Entomology 53 (1):67-75

210. Lyu Z, Wan L-h, Yang Y-q, Tang R, Xu L-z (2016) A checklist of beetles (Insecta, Coleoptera) on pig carcasses in the suburban area of southwestern China: A preliminary study and its forensic relevance. Journal of Forensic and Legal Medicine 41:42-48

211. Mashaly AMA, Al-Mekhlafi FA (2016) Differential Diptera Succession Patterns on Decomposed Rabbit Carcasses in Three Different Habitats. Journal of Medical Entomology 53 (5):1192-1197

212. Metcalf JL, Xu ZZ, Weiss S, Lax S, Van Treuren W, Hyde ER, Song SJ, Amir A, Larsen P, Sangwan N, Haarmann D, Humphrey GC, Ackermann G, Thompson LR, Lauber C, Bibat A, Nicholas C, Gebert MJ, Petrosino JF, Reed SC, Gilbert JA, Lynne AM, Bucheli SR, Carter DO, Knight R (2016) Microbial community assembly and metabolic function during mammalian corpse decomposition. Science 351 (6269):158-162

213. Moffatt C, Heaton V, De Haan D (2016) The distribution of blow fly (Diptera: Calliphoridae) larval lengths and its implications for estimating post mortem intervals. International Journal of Legal Medicine 130 (1):287-297

214. Parry N, Mansell M, Weldon C (2016) Seasonal, locality, and habitat variation in assemblages of carrion-associated Diptera in Gauteng Province, South Africa. Journal of Medical Entomology 53 (6):1322-1329

215. Perez AE, Haskell NH, Wells JD (2016) Commonly Used Intercarcass Distances Appear to Be Sufficient to Ensure Independence of Carrion Insect Succession Pattern. Annals of the Entomological Society of America 109 (1):72-80

216. Weidner LM, Monzon MA, Hamilton GC (2016) Death eaters respond to the dark mark of decomposition day and night: observations of initial insect activity on piglet carcasses. International Journal of Legal Medicine 130 (6):1633-1637

217. Weiss S, Carter DO, Metcalf JL, Knight R (2016) Carcass mass has little influence on the structure of gravesoil microbial communities. International Journal of Legal Medicine 130 (1):253-263

218. Vasconcelos SD, Salgado RL, Barbosa TM, Souza JRB (2016) Diptera of Medico-Legal Importance Associated With Pig Carrion in a Tropical Dry Forest. Journal of Medical Entomology 53 (5):1131-1139

219. Amendt J, Rodner S, Schuch C-P, Sprenger H, Weidlich L, Reckel F (2017) Helicopter thermal imaging for detecting insect infested cadavers. Science & Justice 57 (5):366-372

220. Connor M, Baigent C, Hansen ES (2018) Testing the Use of Pigs as Human Proxies in Decomposition Studies. Journal of Forensic Sciences 63 (5):1350-1355

221. Fancher JP, Aitkenhead-Peterson JA, Farris T, Mix K, Schwab AP, Wescott DJ, Hamilton MD (2017) An evaluation of soil chemistry in human cadaver decomposition islands: Potential for estimating postmortem interval (PMI). Forensic Science International 279:130-139

222. Marais-Werner A, Myburgh J, Meyer A, Nienaber WC, Steyn M (2017) Decomposition patterns of buried remains at different intervals in the Central Highveld region of South Africa. Medicine, Science and the Law 57 (3):115-123

223. Martín-Vega D, Nieto CM, Cifrián B, Baz A, Díaz-Aranda LM (2017) Early colonisation of urban indoor carcasses by blow flies (Diptera: Calliphoridae): an experimental study from central Spain. Forensic Science International 278:87-94

224. Mashaly AMA (2017) Carrion beetles succession in three different habitats in Riyadh, Saudi Arabia. Saudi Journal of Biological Sciences 24 (2):430-435

225. McIntosh CS, Dadour IR, Voss SC (2017) A comparison of carcass decomposition and associated insect succession onto burnt and unburnt pig carcasses. International Journal of Legal Medicine 131 (3):835-845

226. Michaud J-P, Moreau G (2017) Facilitation may not be an adequate mechanism of community succession on carrion. Oecologia 183 (4):1143-1153

227. Niederegger S, Steube X, Tiltmann P, Mall G (2017) Decomposition rate of intact and injured piglet cadavers. Rechtsmedizin 27 (1):8-15

228. Pacheco V, Hans K, VanLaerhoven S (2017) The Relationship Between Surface Area and Volume of Common Blow Fly (Diptera: Calliphoridae) Oviposition Sites and Carrion Body Mass. Journal of Medical Entomology 54 (5):1278-1284

229. Roberts LG, Spencer JR, Dabbs GR (2017) The effect of body mass on outdoor adult human decomposition. Journal of Forensic Sciences 62 (5):1145-1150

230. Scholl K, Moffatt C (2017) Plastic waste sacks alter the rate of decomposition of dismembered bodies within. International Journal of Legal Medicine 131 (4):1141-1147

231. Wang Y, Ma MY, Jiang XY, Wang JF, Li LL, Yin XJ, Wang M, Lai Y, Tao LY (2017) Insect succession on remains of human and animals in Shenzhen, China. Forensic Science International 271:75-86

232. Wang Y, Wang J, Wang Z, Tao L (2017) Insect succession on pig carcasses using different exposure time - A preliminary study in Guangzhou, China. Journal of Forensic and Legal Medicine 52:24-29

233. Weidner LM, Gemmellaro MD, Tomberlin JK, Hamilton GC (2017) Evaluation of bait traps as a means to predict initial blow fly (Diptera: Calliphoridae) communities associated with decomposing swine remains in New Jersey, USA. Forensic Science International 278:95-100

234. Cruise A, Hatano E, Watson DW, Schal C (2018) Comparison of techniques for sampling adult necrophilous insects from pig carcasses. Journal of Medical Entomology 55 (4):947-954

235. Cruise A, Watson DW, Schal C (2018) Ecological succession of adult necrophilous insects on neonate Sus scrofa domesticus in central North Carolina. PloS ONE 13 (4):e0195785

236. Dautartas A, Kenyhercz MW, Vidoli GM, Meadows Jantz L, Mundorff A, Steadman DW (2018) Differential Decomposition Among Pig, Rabbit, and Human Remains. Journal of Forensic Sciences 63 (6):1673-1683

237. Steadman DW, Dautartas A, Kenyhercz MW, Jantz LM, Mundorff A, Vidoli GM (2018) Differential Scavenging Among Pig, Rabbit, and Human Subjects. Journal of Forensic Sciences 63 (6):1684-1691

238. Díaz-Aranda LM, Martín-Vega D, Gómez-Gómez A, Cifrián B, Baz A (2018) Annual variation in decomposition and insect succession at a periurban area of central Iberian Peninsula. Journal of Forensic and Legal Medicine 56:21-31

239. Frątczak-Łagiewska K, Matuszewski S (2018) Resource partitioning between closely related carrion beetles: Thanatophilus sinuatus (F.) and Thanatophilus rugosus (L.)(Coleoptera: Silphidae). Entomologia Generalis 37 (2):143-156

240. von Hoermann C, Jauch D, Kubotsch C, Reichel-Jung K, Steiger S, Ayasse M (2018) Effects of abiotic environmental factors and land use on the diversity of carrion-visiting silphid beetles (Coleoptera: Silphidae): A large scale carrion study. PloS ONE 13 (5):e0196839

241. Knobel Z, Ueland M, Nizio KD, Patel D, Forbes SL (2018) A comparison of human and pig decomposition rates and odour profiles in an Australian environment. Australian Journal of Forensic Sciences:1-16

242. Lee MJ, Voss SC, Franklin D, Dadour IR (2018) Preliminary investigation of aircraft mounted thermal imaging to locate decomposing remains via the heat produced by larval aggregations. Forensic Science International 289:175-185

243. Lutz L, Amendt J, Moreau G (2018) Carcass concealment alters assemblages and reproduction of forensically important beetles. Forensic Science International 291:124-132

244. Mañas-Jordá S, León-Cortés JL, García-García MD, Caballero U, Infante F (2018) Dipteran Diversity and Ecological Succession on Dead Pigs in Contrasting Mountain Habitats of Chiapas, Mexico. Journal of Medical Entomology 55 (1):59-68

245. Marais-Werner A, Myburgh J, Becker PJ, Steyn M (2018) A comparison between decomposition rates of buried and surface remains in a temperate region of South Africa. International Journal of Legal Medicine 132 (1):301-309

246. Pérez-Marcos M, Arnaldos MI, López-Gallego E, Luna A, Khedre A, García MD (2018) An approach for identifying the influence of carcass type and environmental features on sarcosaprophagous Diptera communities. Annales de la Société Entomologique de France (NS) 54 (4):367-380

247. Salimi M, Chatrabgoun O, Akbarzadeh K, Oshaghi M, Falahati MH, Rafizadeh S, Yusuf MA, Rassi Y (2018) Evaluation of Insect Succession Patterns and Carcass Weight Loss for the Estimation of Postmortem Interval. Journal of Medical Entomology 55 (6):1410-1422

248. Shayya S, Dégallier N, Nel A, Azar D, Lackner T (2018) Contribution to the knowledge of Saprinus Erichson, 1834 of forensic relevance from Lebanon (Coleoptera, Histeridae). ZooKeys (738):117

249. Singh B, Minick KJ, Strickland MS, Wickings KG, Crippen TL, Tarone AM, Benbow ME, Sufrin N, Tomberlin JK, Pechal JL (2018) Temporal and Spatial Impact of Human Cadaver Decomposition on Soil Bacterial and Arthropod Community Structure and Function. Frontiers in Microbiology 8:2616

250. Spies MJ, Finaughty DA, Gibbon VE (2018) Forensic taphonomy: Scavenger-induced scattering patterns in the temperate southwestern Cape, South Africa — A first look. Forensic Science International 290:29-35

251. Spies MJ, Gibbon VE, Finaughty DA (2018) Forensic taphonomy: Vertebrate scavenging in the temperate southwestern Cape, South Africa. Forensic Science International 290:62-69

252. Szelecz I, Feddern N, Seppey C, Amendt J, Mitchell E (2018) The importance of Saprinus semistriatus (Coleoptera: Histeridae) for estimating the minimum post-mortem interval. Legal Medicine 30:21-27

253. Szelecz I, Koenig I, Seppey CV, Le Bayon R-C, Mitchell EA (2018) Soil chemistry changes beneath decomposing cadavers over a one-year period. Forensic Science International 286:155-165

1. References to Table 2

1. Schoenly K, Griest K, Rhine S (1991) An experimental field protocol for investigating the postmortem interval using multidisciplinary indicators. Journal of Forensic Sciences 36 (5):1395-1415

2. Schoenly KG, Haskell NH, Hall RD, Gbur JR (2007) Comparative performance and complementarity of four sampling methods and arthropod preference tests from human and porcine remains at the Forensic Anthropology Center in Knoxville, Tennessee. Journal of Medical Entomology 44 (5):881-894

3. Watson EJ, Carlton CE (2003) Spring succession of necrophilous insects on wildlife carcasses in Louisiana. Journal of Medical Entomology 40 (3):338-347

4. Watson EJ, Carlton CE (2005) Insect succession and decomposition of wildlife carcasses during fall and winter in Louisiana. Journal of Medical Entomology 42 (2):193-203

5. Wang Y, Ma MY, Jiang XY, Wang JF, Li LL, Yin XJ, Wang M, Lai Y, Tao LY (2017) Insect succession on remains of human and animals in Shenzhen, China. Forensic Science International 271:75-86

6. Connor M, Baigent C, Hansen ES (2018) Testing the Use of Pigs as Human Proxies in Decomposition Studies. Journal of Forensic Sciences 63 (5):1350-1355

7. Dautartas A, Kenyhercz MW, Vidoli GM, Meadows Jantz L, Mundorff A, Steadman DW (2018) Differential Decomposition Among Pig, Rabbit, and Human Remains. Journal of Forensic Sciences 63 (6):1673-1683

8. Vance GM, VanDyk JK, Rowley WA (1995) A device for sampling aquatic insects associated with carrion in water. Journal of Forensic Sciences 40 (3):479-482

9. Michaud JP, Moreau G (2011) A statistical approach based on accumulated degree-days to predict decomposition-related processes in forensic studies. Journal of Forensic Sciences 56 (1):229-232

10. Hofer IM, Hart AJ, Martín-Vega D, Hall MJ (2017) Optimising crime scene temperature collection for forensic entomology casework. Forensic Science International 270:129-138

11. Matuszewski S (2011) Estimating the pre-appearance interval from temperature in Necrodes littoralis L. (Coleoptera: Silphidae). Forensic Science International 212 (1–3):180-188

12. Matuszewski S (2012) Estimating the Preappearance Interval from Temperature in Creophilus maxillosus L. (Coleoptera: Staphylinidae). Journal of Forensic Sciences 57 (1):136-145

13. Matuszewski S, Szafałowicz M (2013) Temperature-dependent appearance of forensically useful beetles on carcasses. Forensic Science International 229 (1–3):92-99

14. Archer M (2014) Comparative Analysis of Insect Succession Data from Victoria (Australia) Using Summary Statistics versus Preceding Mean Ambient Temperature Models. Journal of Forensic Sciences 59 (2):404-412

15. Matuszewski S, Szafalowicz M, Grzywacz A (2014) Temperature-dependent appearance of forensically useful flies on carcasses. International Journal of Legal Medicine 128 (6):1013-1020

16. Matuszewski S, Mądra A (2015) Factors affecting quality of temperature models for the pre-appearance interval of forensically useful insects. Forensic Science International 247:28-35

17. Matuszewski S, Mądra-Bielewicz A (2016) Validation of temperature methods for the estimation of pre-appearance interval in carrion insects. Forensic Science, Medicine and Pathology 12 (1):50-57

18. Myburgh J, L'Abbe EN, Steyn M, Becker PJ (2013) Estimating the postmortem interval (PMI) using accumulated degree-days (ADD) in a temperate region of South Africa. Forensic Science International 229 (1-3):165 e161-166

19. Lynch-Aird J, Moffatt C, Simmons T (2015) Decomposition Rate and Pattern in Hanging Pigs. Journal of Forensic Sciences 60 (5):1155-1163

20. Nawrocka M, Frątczak K, Matuszewski S (2016) Inter‐Rater Reliability of Total Body Score—A Scale for Quantification of Corpse Decomposition. Journal of Forensic Sciences 61 (3):798-802

21. Keough N, Myburgh J, Steyn M (2017) Scoring of Decomposition: A Proposed Amendment to the Method When Using a Pig Model for Human Studies. Journal of Forensic Sciences 62 (4):986-993

22. Ribéreau-Gayon A, Rando C, Morgan RM, Carter DO (2018) The suitability of visual taphonomic methods for digital photographs: An experimental approach with pig carcasses in a tropical climate. Science & Justice 58 (3):167-176

23. Michaud JP, Moreau G (2009) Predicting the visitation of carcasses by carrion-related insects under different rates of degree-day accumulation. Forensic Science International 185 (1-3):78-83

24. Michaud JP, Moreau G (2013) Effect of variable rates of daily sampling of fly larvae on decomposition and carrion insect community assembly: implications for forensic entomology field study protocols. Journal of Medical Entomology 50 (4):890-897

25. Perez AE, Haskell NH, Wells JD (2014) Evaluating the utility of hexapod species for calculating a confidence interval about a succession based postmortem interval estimate. Forensic Science International 241:91-95

26. Mohr RM, Tomberlin JK (2015) Development and validation of a new technique for estimating a minimum postmortem interval using adult blow fly (Diptera: Calliphoridae) carcass attendance. International Journal of Legal Medicine 129 (4):851-859

27. Perez AE, Haskell NH, Wells JD (2016) Commonly Used Intercarcass Distances Appear to Be Sufficient to Ensure Independence of Carrion Insect Succession Pattern. Annals of the Entomological Society of America 109 (1):72-80

28. Matuszewski S (2017) A general approach for postmortem interval based on uniformly distributed and interconnected qualitative indicators. International Journal of Legal Medicine 131 (3):877-884

29. Mądra‐Bielewicz A, Frątczak‐Łagiewska K, Matuszewski S (2017) Sex‐and Size‐Related Patterns of Carrion Visitation in Necrodes littoralis (Coleoptera: Silphidae) and Creophilus maxillosus (Coleoptera: Staphylinidae). Journal of Forensic Sciences 62 (5):1229-1233

30. Cruise A, Hatano E, Watson DW, Schal C (2018) Comparison of techniques for sampling adult necrophilous insects from pig carcasses. Journal of Medical Entomology 55 (4):947-954

31. VanLaerhoven S (2008) Blind validation of postmortem interval estimates using developmental rates of blow flies. Forensic Science International 180 (2-3):76-80

32. Reibe-Pal S, Madea B (2015) Calculating time since death in a mock crime case comparing a new computational method (ExLAC) with the ADH method. Forensic Science International 248:78-81

33. Weatherbee CR, Pechal JL, Stamper T, Benbow ME (2017) Post-Colonization Interval Estimates Using Multi-Species Calliphoridae Larval Masses and Spatially Distinct Temperature Data Sets: A Case Study. Insects 8 (2):40

34. Pechal JL, Crippen TL, Benbow ME, Tarone AM, Dowd S, Tomberlin JK (2014) The potential use of bacterial community succession in forensics as described by high throughput metagenomic sequencing. International Journal of Legal Medicine 128 (1):193-205

35. Amendt J, Rodner S, Schuch C-P, Sprenger H, Weidlich L, Reckel F (2017) Helicopter thermal imaging for detecting insect infested cadavers. Science & Justice 57 (5):366-372

36. Lee MJ, Voss SC, Franklin D, Dadour IR (2018) Preliminary investigation of aircraft mounted thermal imaging to locate decomposing remains via the heat produced by larval aggregations. Forensic Science International 289:175-185

37. Schultz JJ, Collins ME, Falsetti AB (2006) Sequential monitoring of burials containing large pig cadavers using ground‐penetrating radar. Journal of Forensic Sciences 51 (3):607-616

38. Schultz JJ (2008) Sequential monitoring of burials containing small pig cadavers using ground penetrating radar. Journal of Forensic Sciences 53 (2):279-287

39. Salsarola D, Poppa P, Amadasi A, Mazzarelli D, Gibelli D, Zanotti E, Porta D, Cattaneo C (2015) The utility of ground-penetrating radar and its time-dependence in the discovery of clandestine burials. Forensic Science International 253:119-124

40. Healy CA, Schultz JJ, Parker K, Lowers B (2015) Detecting Submerged Bodies: Controlled Research Using Side-Scan Sonar to Detect Submerged Proxy Cadavers. Journal of Forensic Sciences 60 (3):743-752

41. Pahor K, Olson G, Forbes SL (2013) Post-mortem detection of gasoline residues in lung tissue and heart blood of fire victims. International Journal of Legal Medicine 127 (5):923-930
